# The alpha-1 subunit of the Na^+^,K^+^-ATPase (ATP1A1) is required for macropinocytic entry of respiratory syncytial virus (RSV) in human respiratory epithelial cells

**DOI:** 10.1371/journal.ppat.1007963

**Published:** 2019-08-05

**Authors:** Matthias Lingemann, Thomas McCarty, Xueqiao Liu, Ursula J. Buchholz, Sonja Surman, Scott E. Martin, Peter L. Collins, Shirin Munir

**Affiliations:** 1 RNA Viruses Section, Laboratory of Infectious Diseases, National Institute of Allergy and Infectious Diseases, National Institutes of Health, Bethesda, Maryland, United States of America; 2 Institut für Mikrobiologie, Technische Universität Braunschweig, Braunschweig, Germany; 3 Division of Pre-Clinical Innovation, National Center for Advancing Translational Sciences, Rockville, Maryland, United States of America; Harvard Medical School, UNITED STATES

## Abstract

Human respiratory syncytial virus (RSV) is the leading viral cause of acute pediatric lower respiratory tract infections worldwide, with no available vaccine or effective antiviral drug. To gain insight into virus-host interactions, we performed a genome-wide siRNA screen. The expression of over 20,000 cellular genes was individually knocked down in human airway epithelial A549 cells, followed by infection with RSV expressing green fluorescent protein (GFP). Knockdown of expression of the cellular ATP1A1 protein, which is the major subunit of the Na^+^,K^+^-ATPase of the plasma membrane, had one of the strongest inhibitory effects on GFP expression and viral titer. Inhibition was not observed for vesicular stomatitis virus, indicating that it was RSV-specific rather than a general effect. ATP1A1 formed clusters in the plasma membrane very early following RSV infection, which was independent of replication but dependent on the attachment glycoprotein G. RSV also triggered activation of ATP1A1, resulting in signaling by c-Src-kinase activity that transactivated epidermal growth factor receptor (EGFR) by Tyr845 phosphorylation. ATP1A1 signaling and activation of both c-Src and EGFR were found to be required for efficient RSV uptake. Signaling events downstream of EGFR culminated in the formation of macropinosomes. There was extensive uptake of RSV virions into macropinosomes at the beginning of infection, suggesting that this is a major route of RSV uptake, with fusion presumably occurring in the macropinosomes rather than at the plasma membrane. Important findings were validated in primary human small airway epithelial cells (HSAEC). In A549 cells and HSAEC, RSV uptake could be inhibited by the cardiotonic steroid ouabain and the digitoxigenin derivative PST2238 (rostafuroxin) that bind specifically to the ATP1A1 extracellular domain and block RSV-triggered EGFR Tyr845 phosphorylation. In conclusion, we identified ATP1A1 as a host protein essential for macropinocytic entry of RSV into respiratory epithelial cells, and identified PST2238 as a potential anti-RSV drug.

## Author summary

RSV continues to be the most important viral cause of severe bronchiolitis and pneumonia in infants and young children, and also has a substantial impact in the elderly. It is estimated to claim the lives of ~118,000 children under five years of age annually. No vaccine or antiviral drug suitable for general use is available. The involvement of host factors in RSV infection and replication is not well understood, but this knowledge might lead to intervention strategies to prevent infection. Using a genome-wide siRNA screen to knock down the expression of over 20,000 individual cellular genes, we identified ATP1A1, the major subunit of the Na^+^,K^+^-ATPase, as an important host protein for RSV entry. We showed that ATP1A1 activation by RSV resulted in transactivation of EGFR by Src-kinase activity, resulting in the uptake of RSV particles into the host cell through macropinocytosis. We also showed that the cardiotonic steroid ouabain and the synthetic digitoxigenin derivative PST2238, which bind specifically to the extracellular domain of ATP1A1, significantly reduced RSV entry. Taken together, we describe a novel ATP1A1-enabled mechanism used by RSV to enter the host cell, and describe candidate antiviral drugs that block this entry.

## Introduction

Human respiratory syncytial virus (RSV) is the leading viral cause of severe lower respiratory tract infections in infants and young children worldwide [1]. RSV-associated acute lower respiratory infection of children younger than 5 years of age causes ~118,000 deaths worldwide annually [2]. So far, no licensed RSV vaccine or effective antiviral drug is available, although a number of vaccine and drug candidates are under development. Understanding the fundamental mechanisms of virus-host interactions and identifying factors critical for RSV infection might lead to the development of new antiviral drugs.

RSV has a single stranded, non-segmented, negative sense RNA genome and belongs to the genus *Orthopneumovirus* of the family *Pneumoviridae* within the order *Mononegavirales* [3]. The genome is approximately 15.2 kb long and contains 10 genes that encode 11 proteins, namely (in 3’ to 5’ genomic order) the nonstructural proteins NS1 and NS2; nucleocapsid (N); phosphoprotein (P); matrix protein (M); small hydrophobic (SH), attachment (G), and fusion (F) glycoproteins; M2-1 and M2-2 proteins that are encoded by the two overlapping open reading frames in the M2 gene; and large polymerase protein (L) [4]. The envelope glycoproteins G and F mediate viral attachment and fusion, for entry into the host cell, while SH forms ion channels whose role in infection remains unclear. SH and G are not essential for virus replication in immortalized cell lines [5–7], but G is important for efficient replication *in vivo* [8].

A number of host proteins and pathways have been suggested to play roles in RSV attachment and entry, but a detailed understanding remained elusive. For instance, it was shown that RSV utilizes lipid rafts in cholesterol-rich microdomains on the cell surface known as caveolae as a docking platform for entry [9]. Cell surface glycosaminoglycans (GAGs) appear to be important in RSV attachment to immortalized cell lines [10], but GAGs do not appear to be present on the apical surfaces of primary epithelial cells and so may not play an important role *in vivo*. The G protein contains a CX3C motif reported to mediate cell attachment by binding to the chemokine receptor CX3CR1 [11, 12]. Nucleolin also has been reported as a cellular receptor for RSV that is bound by the F protein [13]. Epidermal growth factor receptor (EGFR) signaling has been postulated to play a role in triggering macropinocytic uptake of RSV [14], but how this occurs was unknown. It remained unknown if EGFR alone is sufficient or requires other factors to initiate signaling, or if EGFR and its associated signaling are somehow physically linked with caveolae. While RSV entry generally has been thought to involve fusion between the viral envelope and the plasma membrane, new evidence suggested either of two additional, different uptake pathways, namely EGFR-triggered macropinocytosis [14] and clathrin-mediated endocytosis [15]. It is unclear if one or both are involved.

We describe an *in vitro* genome-wide siRNA screen that was performed to identify cellular proteins with presumptive roles in RSV infection. One of the proteins identified by this screen was described previously [16]. Another protein that was identified is ATP1A1 (GenBank ID: 476), the α-subunit of the Na^+^,K^+^-ATPase complex located in the plasma membrane. This complex contains in addition a β-subunit, and usually also a γ-subunit (also known as the FXYD subunit) [17, 18]. ATP1A1 is the major subunit and contains ten transmembrane helices that embed the protein complex in the plasma membrane and form the ion channel. The β and FXYD subunits are important for the ion transport properties of the Na^+^,K^+^-ATPase and also stabilize the complex [19]. Humans express three additional α-isoforms beside ATP1A1 (ATP1A2, ATP1A3, and ATP1A4). The expression profile of the four isoforms is cell type-dependent, with ATP1A1 being expressed ubiquitously and being the predominant isoform in A549 cells [20] (minor amounts of ATP1A3 have been found in A549 cells, but are 50-fold reduced compared to ATP1A1 [20]). In the ciliated epithelial cells lining the nasopharynx and the bronchi of both mice and humans, Na^+^,K^+^-ATPase bearing ATP1A1 is present in greater abundance on the basolateral surfaces and in lower abundance on the apical surface; it also is readily detected in human alveolar cells including on the luminal surface [21–23].

Na^+^,K^+^-ATPase plays a major role in ion transport, maintaining electrolyte and fluid balance. In addition, a subpopulation of Na^+^,K^+^-ATPase that is localized in caveolae [24, 25] uniquely can engage in signal transduction, via the ATP1A1 subunit [17, 26–28]. Na^+^,K^+^-ATPase, bearing the ATP1A1 subunit, has been well-characterized as the sole receptor for cardiotonic steroids (CTS) such as ouabain, which are its sole known agonists initiating signaling. Ouabain has been reported in humans as an endogenous hormone-like agent that contributes to the regulation of blood pressure, among other things, via ATP1A1 signaling [29].

ATP1A1 does not possess a known cytoplasmic signaling domain, but it interacts through its cytoplasmic tail with the cellular kinase c-Src [30]. ATP1A1 initiates signaling by conferring a conformational change to c-Src that exposes its kinase domain, leading to activation through autophosphorylation of c-Src at tyrosine 418 [30]. This can trigger several different signaling pathways, which can lead to the induction of endocytosis by more than one mechanism. For example, activated c-Src can mediate phosphorylation and activation of EGFR in an EGF-independent manner [31, 32], which can induce macropinocytosis [31], similar to the well-characterized EGF-induced macropinocytosis [33, 34]. Activated c-Src also can trigger signaling through the PI3K pathway to induce clathrin-mediated endocytosis [17, 26]. Incidentally, this results in removal of Na^+^,K^+^-ATPase from the plasma membrane for degradation in the lysosome [35, 36], resulting in decreased ion channel activity and increased blood pressure. This can be reversed by a synthetic digitoxigenin derivative called PST2238 (or rostafuroxin), which competitively inhibits ouabain binding and signaling [37] and is being evaluated as a therapeutic to lower this kind of hypertension [38].

Here, we report a novel role for ATP1A1 signaling in enabling RSV entry into human airway epithelial cells. We demonstrate that RSV induces the signaling function of ATP1A1 to enable cell entry by a mechanism that is dependent on the activation of c-Src and EGFR. We also show that this signaling results in the induction of macropinocytosis, which appears to be a major route for uptake of RSV into the cell, with membrane fusion presumably occurring in the macropinosomes. We also provide evidence that RSV-induced ATP1A1 activation and signaling occur in the caveolae and can be inhibited by cholesterol depletion. The cardiotonic steroid ouabain or the digitoxigenin derivative PST2238 inhibit RSV-triggered ATP1A1 activation, prevent RSV entry, and thus could serve as a target for antiviral drug development.

## Results

### Knockdown of ATP1A1 expression by siRNA transfection

We performed a genome-wide high-throughput siRNA screen in which the expression of ~ 21,500 cellular genes was individually knocked down in human airway epithelial A549 cells by three individual siRNAs per gene, followed by infection with recombinant RSV expressing enhanced green fluorescent protein (RSV-GFP). Knockdowns that reduced GFP expression at 48 h post-infection (p.i.) with minimal effect on cell viability indicated a presumptive role for that protein in RSV replication.

**Fig 1:**
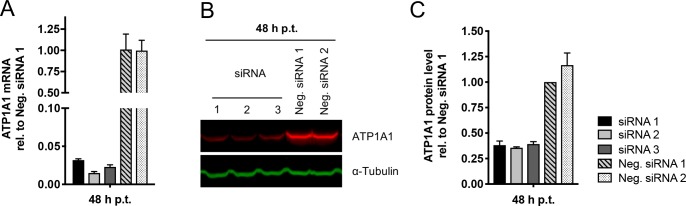
ATP1A1 knock down by siRNA transfection. A549 cells were transfected individually with siRNAs 1, 2, and 3 targeting the ATP1A1 mRNA, as well as with scrambled negative controls Neg. siRNA 1 and 2 that have no known target in human cells. Cells were harvested 24, 48 and 72 h post transfection (p.t.) and the ATP1A1 mRNA and protein levels were quantified. **(A) Relative quantification of ATP1A1 mRNA.** Total RNA was isolated, reverse transcribed, and quantified by an ATP1A1-specific TaqMan Assay. Values were normalized to 18S rRNA and expressed as mean values relative to Neg. siRNA 1 assigned the value of 1.0, with error bars indicating the standard deviation of three independent experiments with three replicate reactions each. **(B) Western blot analysis of ATP1A1 expression.** Cells were lysed at 48 h p.t. and subjected to Western blotting with an anti-ATP1A1 rabbit monoclonal antibody (MAb) (ab76020) and a corresponding infrared dye 680RD-conjugated goat anti-rabbit secondary antibody. Alpha-tubulin was used as a loading control and was detected by an anti-alpha-tubulin mouse MAb and an infrared dye 800CW-conjugated goat anti-mouse secondary antibody. A representative blot is shown. **(C) Quantification of ATP1A1 Western blots**. Western blot analyses of three independent experiments, as described in Part B, were quantified, normalized to alpha-tubulin, and reported relative to Neg. siRNA 1 assigned the value of 1.0, with error bars indicating the standard deviation.

ATP1A1 was among the genes with the strongest effect on RSV-GFP (S1 Table) which was confirmed with additional, different siRNAs. Three siRNAs with the greatest effect on ATP1A1 expression and RSV infection were used for further experiments, and in addition two different scrambled siRNAs (Neg. siRNA 1 and 2) were included as negative controls. To confirm the efficiency of knockdown of ATP1A1 mRNA, A549 cells were transfected with this set of five siRNAs. ATP1A1 mRNA level and the expression of ATP1A1 protein was quantified at different time points post transfection by TaqMan assay and Western blotting, respectively, and reported relative to Neg. siRNA1 at 48 h p.t. (Fig 1); and time course over 72 h (S1 Fig). At 48 h post transfection (p.t.), the level of ATP1A1 mRNA was reduced to below 5% compared to Neg. siRNA 1 (Fig 1A) that resulted in a reduction of the ATP1A1 protein expression at 48 h p.t. to 39% (siRNA1 and 3) and 35% (siRNA2) (Fig 1C) and did not show any further reduction at 72 h p.t. (S1 Fig).

The transfected cells showed no visible cytotoxicity or morphological changes over the period of 72 h. For more sensitive evaluation, cellular ATP, which reflects cell viability, was measured in cell lysates at 72 h p.t. The ATP1A1 siRNA knockdown showed only minimal reductions in cell viability (S1D Fig).

### ATP1A1 knockdown reduces RSV infection

**Fig 2:**
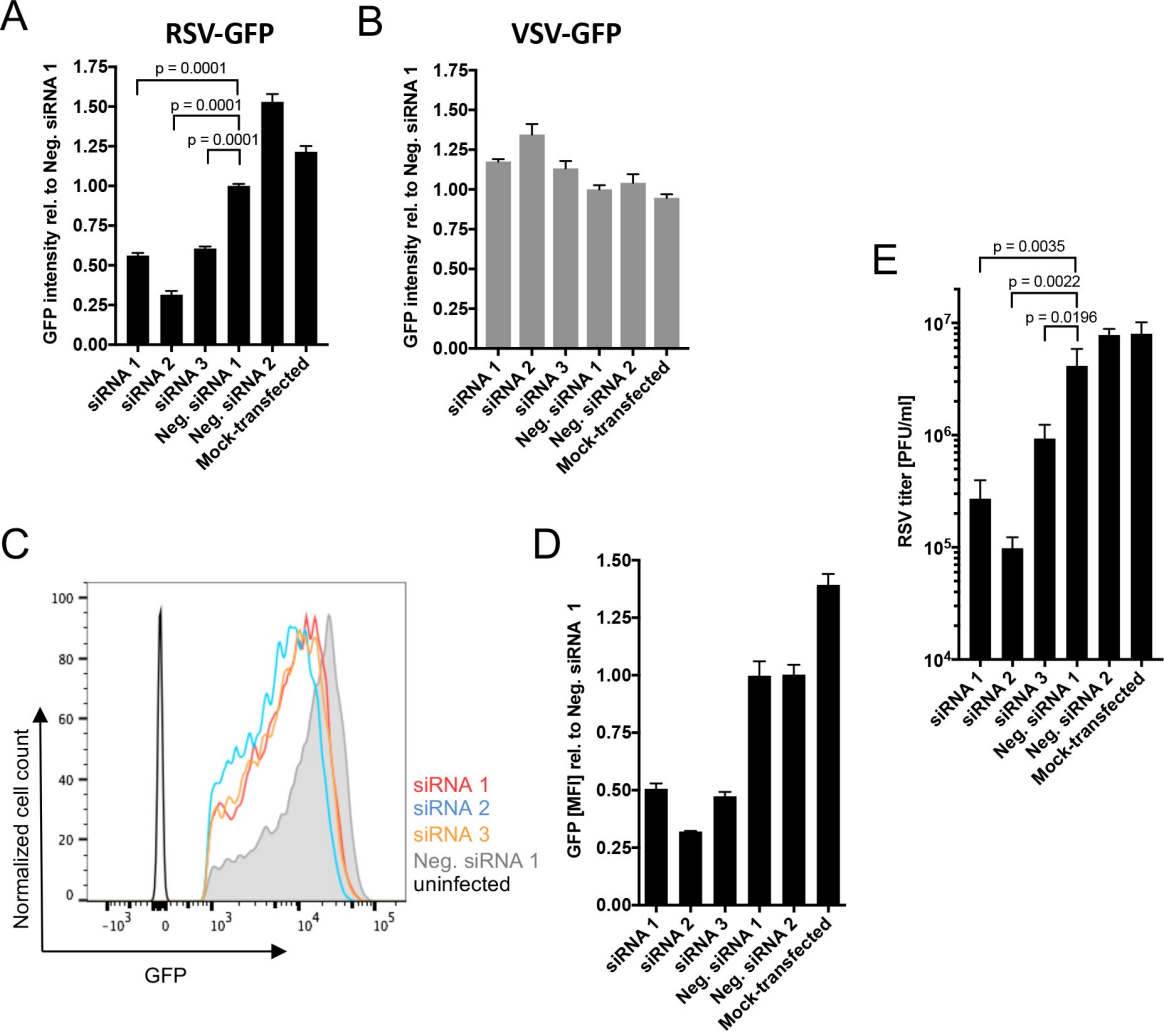
**Effect of ATP1A1 knockdown on infection by RSV-GFP (A, C, D) and VSV-GFP (B).** A549 cells were transfected with the indicated siRNAs and 48 h later were infected with 1 PFU/cell RSV-GFP (A, C, D) or 0.5 PFU/cell VSV-GFP (B). **(A and B) Effects on RSV and VSV**. Following infection with RSV-GFP (A) or VSV-GFP (B), the cells were incubated for 17 h, and the GFP intensity of the total area of each well of A549 cells was quantified by scanning with an ELISA reader and is expressed relative to cells infected with the same virus for which the transfected siRNA was Neg. siRNA 1. **(C and D) Flow cytometry analysis of RSV-GFP expression.** Following infection with RSV-GFP, the cells were incubated for 24 h, and GFP expression of single, live, GFP^+^ cells was quantified by flow cytometry, with cells from an untransfected, uninfected well included for reference. The plot (C) shows cell count versus GFP intensity, and the histogram (D) shows GFP MFI relative to cells infected with the same virus for which the transfected siRNA was Neg. siRNA 1. **(E) Titer of progeny RSV-GFP.** Following infection with RSV-GFP (MOI = 1 PFU/cell), the cells were incubated for 24 h, and the cells and media were harvested together (Materials and Methods) and the yield of RSV-GFP was determined by plaque titration on Vero cells. All data in A-D are derived from at least three independent experiments and shown as mean values with error bars indicating the standard deviation. The statistical significance of difference was determined by one-way analysis of variance with Dunnett’s multiple comparison post-test and p-values are shown for each comparison.

A549 cells were transfected with the panel of siRNAs targeting ATP1A1, and 48 h later the cells were infected with RSV-GFP at an MOI of 1 plaque forming unit (PFU)/cell. The efficiency of virus infection and replication were evaluated by GFP expression quantified by ELISA reader (Fig 2A) and flow cytometry (Fig 2C and 2D). By ELISA reader, all three ATP1A1-specific siRNAs reduced the amount of GFP expression by about 50 to 75% compared to Neg. siRNA 1 (Fig 2A). This level of reduction was substantial given that the residual level of ATP1A1 expression remained 35% or greater, as was shown in Fig 1C. We also investigated effects on infection with vesicular stomatitis virus expressing GFP (VSV-GFP). ATP1A1 knockdown had no effect on GFP expression by VSV-GFP (Fig 2B). This indicated that the reduction in GFP expression observed with RSV-GFP was specific to RSV, did not affect VSV, and was not due to some general effect on cellular functions. Analysis of cells infected with RSV-GFP (MOI = 1.0 PFU/cell) by flow cytometry 24 h p.i. showed that knockdown of ATP1A1 resulted in a broad reduction in GFP expression in the infected-cell population rather than a reduction in the number of GFP-expressing cells (Fig 2C and 2D).

The effect of transfection with the panel of siRNAs on the production of progeny RSV-GFP were assessed 24 h p.i. The infected cells were collected by scraping, vortexed to release cell-associated virus, and pelleted by centrifugation. Virus titers in the clarified supernatants were quantified by plaque titration on Vero cells (Fig 2E). This showed that, with ATP1A1 knockdown, RSV titers were reduced between 5- (siRNA3) and 42-fold (siRNA2) compared to Neg. siRNA 1, an effect that was even more dramatic than the reduction in GFP expression described above (Fig 2E versus 2A and 2D). ATP1A1 siRNA 2 showed the strongest effect against both GFP expression and virus production.

### ATP1A1 forms clusters early during RSV infection independent of viral gene transcription or replication

**Fig 3:**
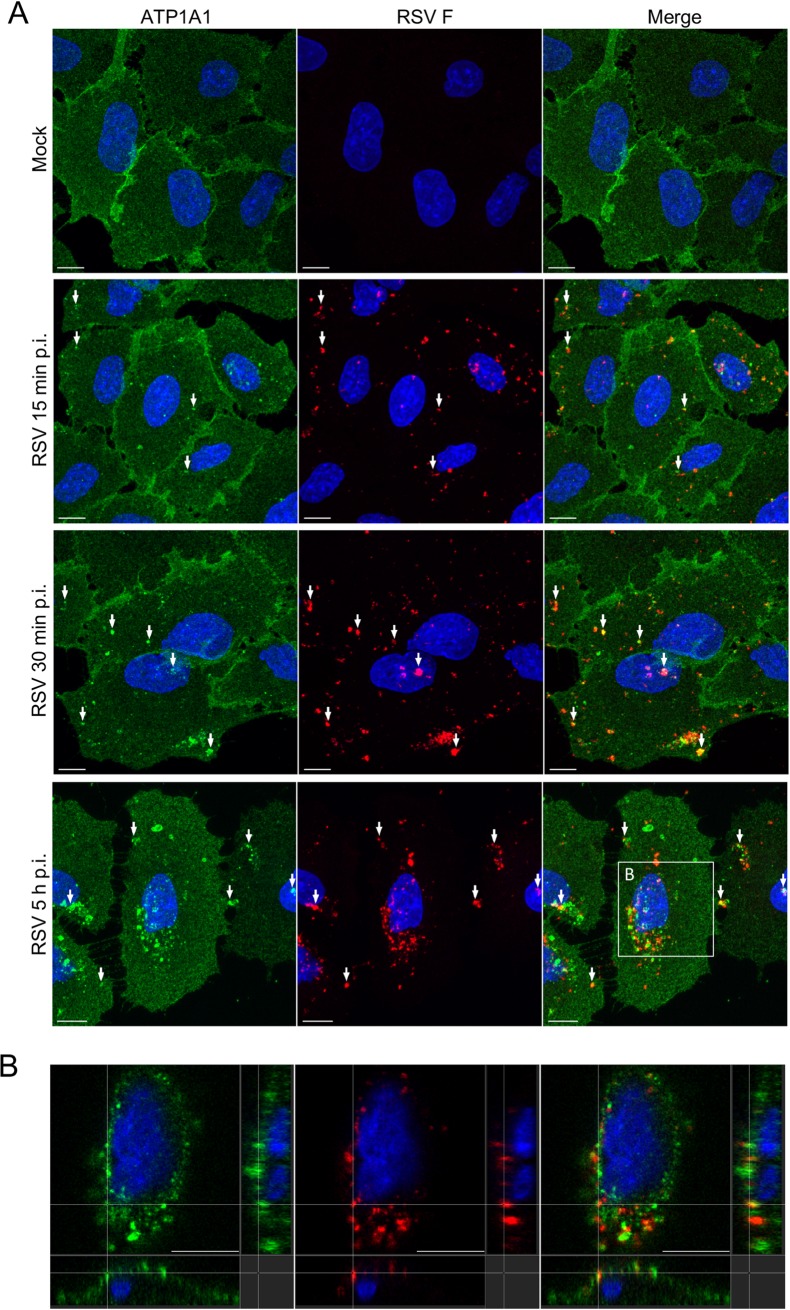
RSV infection triggers ATP1A1 clustering. **(A)** A549 cells were inoculated with wt RSV (MOI = 5 PFU/cell). Cells were fixed at different time points p.i. with 4% PFA, permeabilized with 0.1% TritonX-100 and stained for ATP1A1 (green) with an anti-ATP1A1 MAb (ab76020) and AF488-conjugated donkey anti-rabbit secondary antibody. RSV F (red) was detected with anti-RSV F mouse MAb #1129 [72] and an AF594-conjugated anti-mouse secondary antibody. The cell nuclei were stained with DAPI (blue). Images (z-stacks) were acquired on a Leica SP5 confocal microscope, with a 63x objective (NA 1.4) and a zoom of 3.0. Arrows indicate examples of co-localization of ATP1A1 and RSV F. Scale bar 10 μm. **(B)** Cross section of the marked area of the RSV 5 h p.i. image of Fig 3A. The entire cross-section in ZY-view of this image is shown in S1 Movie. Scale bar 10 μm.

**Fig 4:**
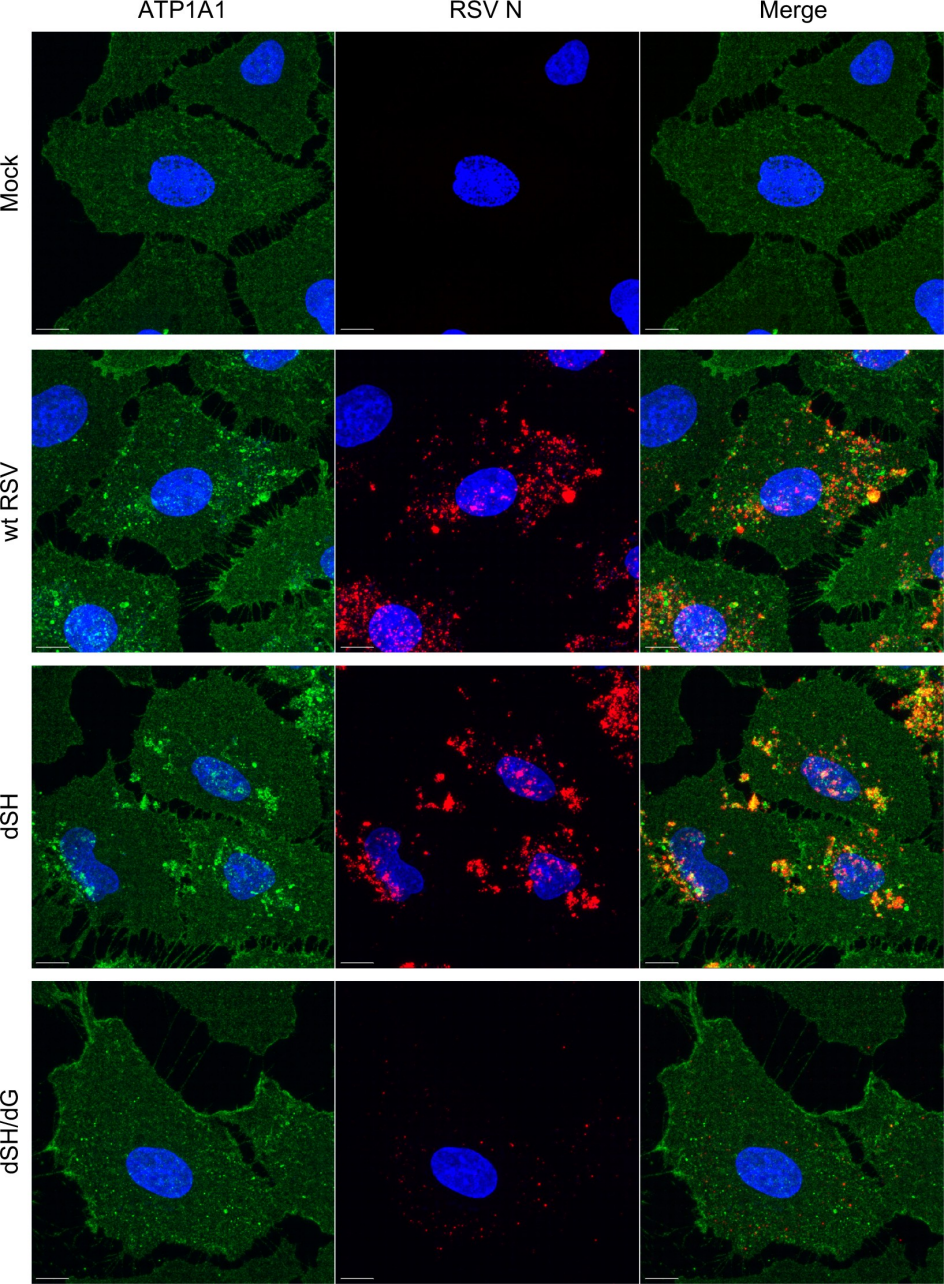
RSV G is required for ATP1A1 clustering. A549 cells were inoculated with wt RSV, rgRSV-dSH or rgRSV-dSH/dG (MOI = 10 PFU/cell) and incubated for 5 h. Cells were fixed with 4% PFA and subjected to immunofluorescence staining, as described for Fig 3. ATP1A1 (green) was detected by an anti-ATP1A1 rabbit MAb (ab76020) and AF568-conjugated donkey anti-rabbit secondary antibody. RSV N (red) was detected by an anti-RSV N mouse MAb (ab94806) and an AF647-conjugated donkey anti-mouse secondary antibody. The cell nuclei were stained with DAPI (blue). Images (z-stacks) were acquired on a Leica SP8 confocal microscope, with a 63x objective (NA 1.4) and a zoom of 3.0. Scale bar 10 μm.

A549 cells were infected with wt RSV (MOI = 5 PFU/cell), fixed at various times p.i., and subjected to immunofluorescence staining for ATP1A1 and RSV F protein. In mock-treated cells, ATP1A1 was homogeneously distributed on the plasma membrane (Fig 3A, top panel). Following infection with wt RSV, clusters of ATP1A1 were observed as early as 15 min p.i. (Fig 3A, second panel), whereas these clusters were not evident in uninfected cells (Fig 3A, top panel). With time, the ATP1A1 clusters became more prominent and numerous, as shown for 30 min and 5 h p.i. (Fig 3A, third and fourth panel). All shown timepoints are very early in the RSV replication cycle. Some ATP1A1 clusters, but not all, partially co-localized with RSV F protein (Fig 3A, indicated by arrows). The localization of clustered ATP1A1 in close proximity to RSV F became more noticeable at later time points such as 5 h p.i. (Fig 3A, bottom panel). Localization of ATP1A1 clusters close to RSV N protein also could be observed (Fig 4 and S2 Fig): co-localization with both F and N suggested that the RSV-specific staining most likely reflects enveloped virions (which had not yet fused). Similar clustering of ATP1A1 and RSV N protein also was observed for UV-inactivated RSV (S2 Fig), indicating that the staining largely involved pre-formed proteins from the incoming virus, and that clustering does not require transcription of the complete viral genome, viral RNA replication, or virus replication.

Cross-sections (Fig 3B) and a ZY-view movie (S1 Movie) of images of RSV-infected A549 cells 5 h p.i. indicate that the ATP1A1 clustering occurred at the cell surface and was localized close to the RSV virions. Given the very early appearance of ATP1A1 clusters, independent of viral transcription or replication, we hypothesized that ATP1A1 might be involved in an early step of infection such as viral entry.

### RSV G protein is required for ATP1A1 clustering

As another means of exploring early events in RSV infection, we investigated whether RSV mutants bearing the deletion of the SH protein (dSH) or the combined deletion of SH and the attachment G glycoprotein (dSH/dG) retained the ability to trigger the clustering of ATP1A1 (deletion of RSV F could not be investigated because it abrogates infectivity). A549 cells were infected with wt RSV, RSV dSH, or RSV dSH/dG (MOI = 10 PFU/cell) and incubated for 5 h. Cells were fixed, permeabilized and immunostained with antibodies specific to ATP1A1 (green) and RSV N (red) (Fig 4). Wt RSV was included as a reference and showed increased cluster formation compared to Fig 3, which may have been due to the increased MOI of 10 PFU/cell compared to 5 PFU/cell. The RSV dSH virus induced clustering that was very similar to that with wt RSV, indicating that deletion of the SH protein seemed to have no effect on ATP1A1 clustering. On the other hand, the dSH/dG virus did not induce any ATP1A1 clustering, and the viral particles, stained for RSV N in red, were reduced in amount and were much more dispersed as compared to wt RSV and the dSH virus. The lack of ATP1A1 cluster formation with the dSH/dG virus suggested that RSV G protein is involved in triggering ATP1A1 clustering.

### Ouabain and PST2238 inhibit RSV infection

The clustering of ATP1A1 in response to RSV exposure suggested that its signaling function might play a role in RSV infection. For example, ATP1A1 signaling cascades can result in the induction of endocytosis including clathrin-mediated or caveolin-mediated endocytosis, or macropinocytosis, which could be involved in RSV entry. The only known agonists for ATP1A1 signaling are CTS such as ouabain, which activate non-receptor tyrosine kinase Src-mediated signaling pathways [29]. The synthetic digitoxigenin derivative PST2238 is a competitive inhibitor of ouabain that inhibits ouabain binding and signaling [37]. Therefore, we tested ouabain and PST2238 for their effects on RSV infection.

**Fig 5:**
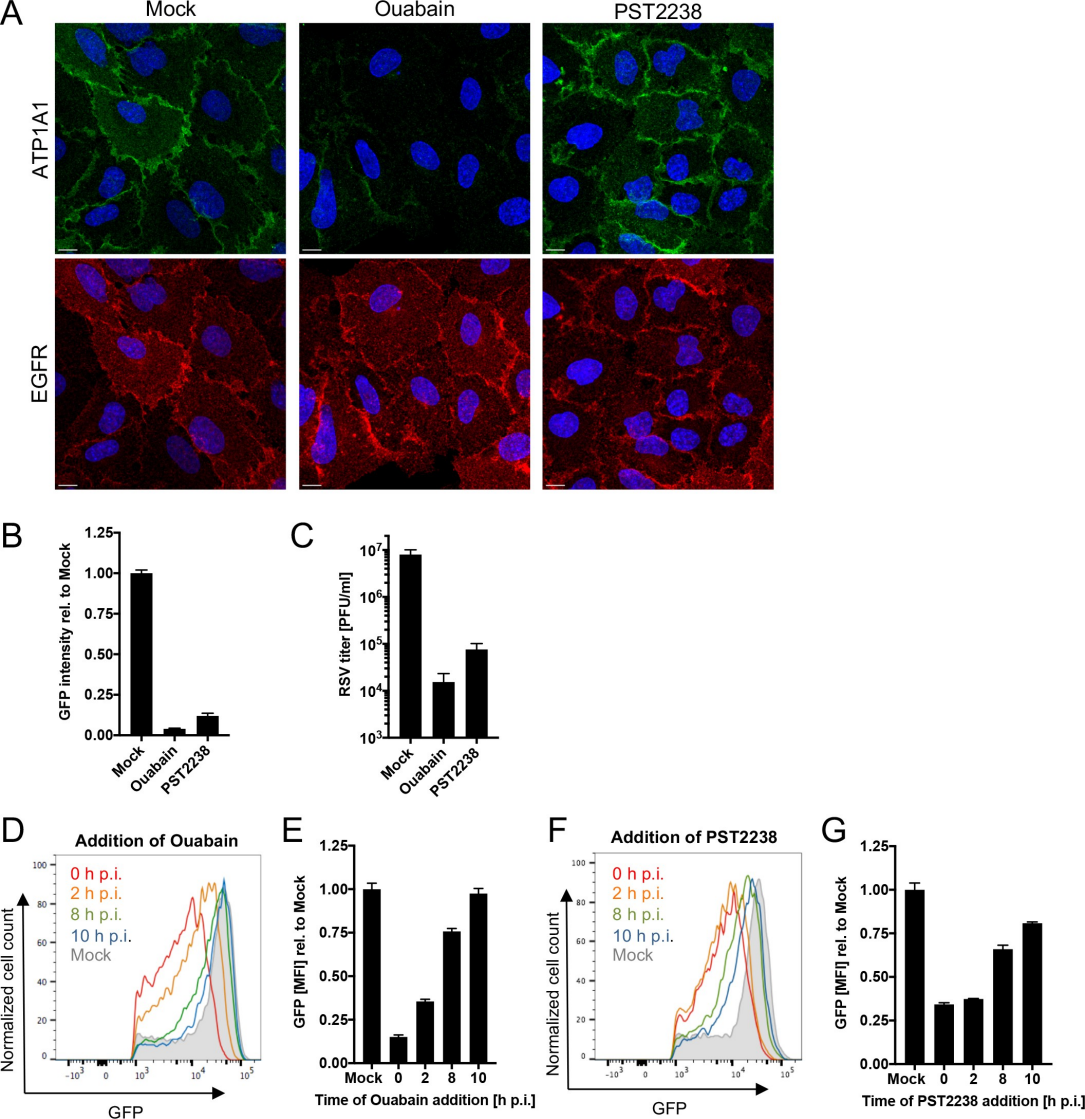
Effect of ouabain and PST2238 on RSV infection. **(A) ATP1A1 and EGFR expression in A549 cells treated with Ouabain or PST2238.** Uninfected A549 cells were treated for 24 h with either 25 nM ouabain or 20 μM PST2238 and subjected to an immunofluorescence staining. ATP1A1 (green) was detected by an anti-ATP1A1 rabbit MAb (ab76020) and an AF488-conjugated donkey anti-rabbit secondary antibody. EGFR (red) was detected by an anti-EGFR rat MAb (ab231) and an AF647-conjugated goat anti-rat secondary antibody. The cell nuclei were stained with DAPI (blue). Scale bar 10 μm. **(B and C) Inhibitory effect on RSV infection.** A549 cells pre-treated for 16 h with either 25 nM ouabain or 20 μM PST2238 were inoculated with RSV-GFP (MOI = 1 PFU/cell). Cells were incubated for 17 h and infectivity was quantified by: (B) GFP signal of the total well, scanned by an ELISA reader and reported relative to mock-treated infected cells as 1.0; and (C) virus titer determined by plaque titration on Vero cells 24 h p.i. **(D—G) Time-of-drug-addition experiment.** A549 cells infected with RSV-GFP (MOI = 3 PFU/cell) were changed to medium containing 25 nM ouabain (D, E) or 20 μM PST2238 (F, G) at the indicated times post infection. Cells were harvested 24 h p.i. and GFP intensity was quantified by flow cytometry of live, single, GFP+ cells. The MFI of GFP+ cells was quantified and expressed relative to mock-treated, RSV-infected cells (E, G).

Serial dilutions of ouabain and PST2238 were evaluated for cytotoxicity on A549 cells (S3C and S3D Fig), and we selected concentrations for ouabain (25 nM) and PST2238 (20 μM) that had less than 20% reduction in cell viability 24 h post treatment, which was the longest treatment period for these studies. The effects of ouabain and PST2238 on ATP1A1 and EGFR expression were analyzed by immunofluorescence microscopy (Fig 5A) using antibodies specific for ATP1A1 and EGFR. EGFR was evaluated in addition to ATP1A1 because it previously was shown to be important for RSV entry [14] and is closely associated with the ATP1A1 signalosome [31, 32]. In mock-treated cells, ATP1A1 and EGFR had homogeneous membrane distributions (Fig 5A, left column). After 24 h treatment with ouabain, the ATP1A1 level was greatly reduced (Fig 5A, middle column, top panel)–presumably due to the removal of cell-surface Na^+^,K^+^-ATPase by clathrin-mediated endocytosis induced by ATP1A1 signaling [35, 36]—while EGFR expression and localization appeared unchanged (Fig 5A, middle column, bottom panel). On the other hand, PST2238 (Fig 5A, right panel) had no discernible effect on the expression and localization of ATP1A1 or EGFR: this drug does not cause removal of ATP1A1 because it does not induce ATP1A1 signaling and endocytosis.

To evaluate possible effects on RSV infection, A549 cells were pre-treated for 16 h with ouabain or PST2238, inoculated with RSV-GFP (MOI = 1 PFU/cell) and incubated with the compounds present throughout. RSV infection was evaluated by (i) GFP expression 17 h p.i. (Fig 5B), and (ii) the yield of progeny RSV harvested 24 h p.i., quantified by plaque assay on Vero cells (Fig 5C). Both methods correlated well, and demonstrated a reduction in RSV replication for both compounds that was greater than that achieved with the ATP1A1-specific siRNAs (Fig 2). Ouabain had the strongest effect: it reduced viral GFP expression by 96% and virus yield by almost 3.0 log_10_ compared to infected cells that did not receive the drug, and the corresponding reductions observed with PST2238 were 89% (GFP reduction) and 2.0 log_10_ (reduction in viral yield).

To investigate the stage of RSV infection that is inhibited by the compounds, a “time of addition” experiment was performed. A549 cells were infected with RSV-GFP (MOI = 3 PFU/cell), and at different times p.i. ouabain (Fig 5D and 5E) or PST2238 (Fig 5F and 5G) was added. Cells were incubated for a total of 24 h p.i. and the viral GFP expression intensity of single, live cells was analyzed by flow cytometry. For both compounds, the strongest inhibitory effect was observed when the inhibitor was added simultaneously with RSV-GFP at 0 h, resulting in 85% and 66% reduction of GFP expression by ouabain and PST2238, respectively (Fig 5D–5G). The inhibition of infection continued to diminish and was almost completely lost when the drugs were added at 10 h p.i. These results suggest a role for ATP1A1 early in infection, such as uptake and entry.

We also investigated whether PST2238 treatment had any effect on the clustering of ATP1A1 that was noted above in response to RSV. A549 cells were pretreated with PST2238 as described above and visualized by confocal microscopy. PST2238 treatment had no apparent effect on ATP1A1 clustering. This indicates that clustering of ATP1A1 did not depend on signaling from ATP1A1 and occurs prior to the step blocked by PST2238.

### Src-kinase activity is required for RSV entry

**Fig 6:**
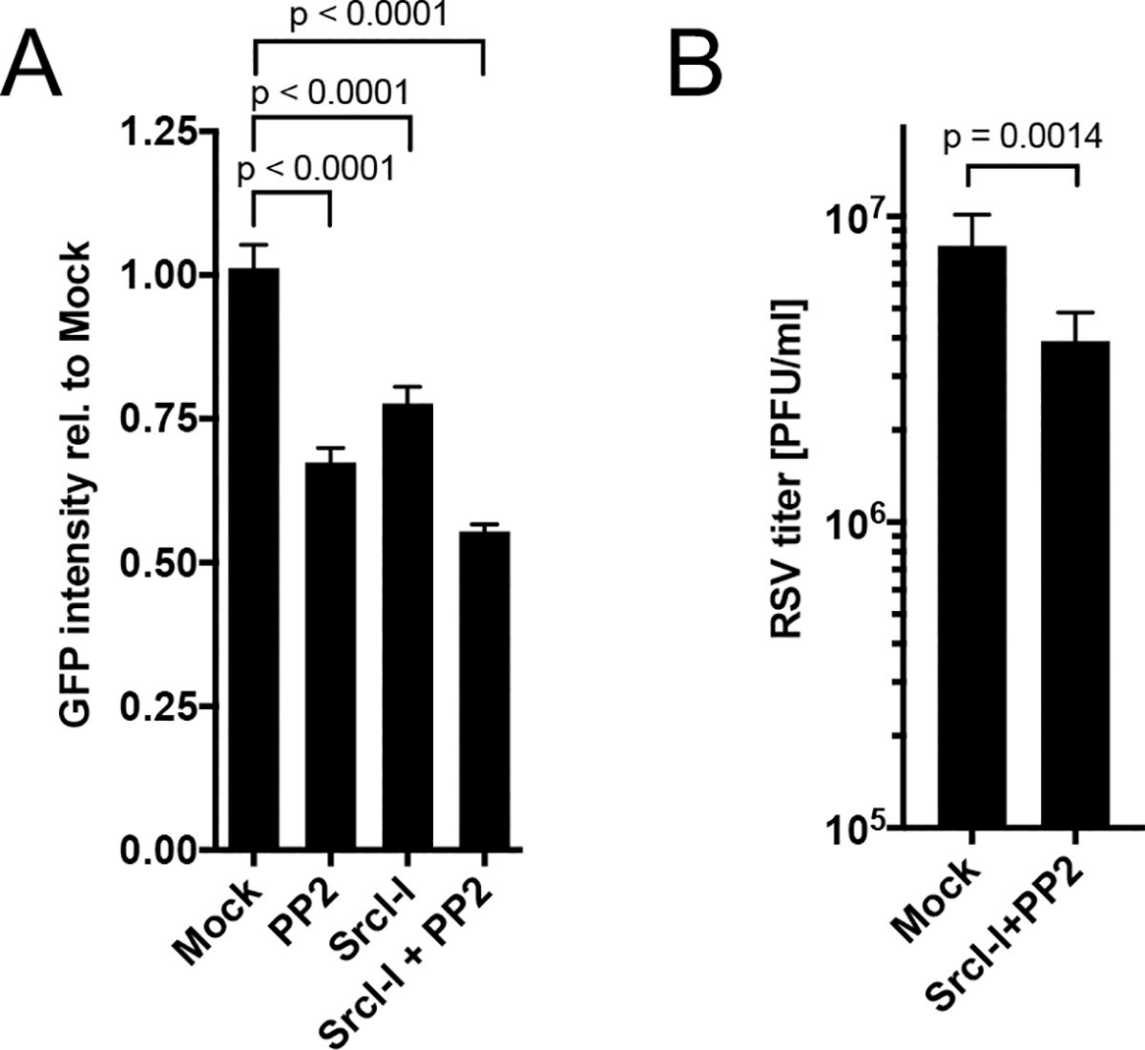
Src-kinase activity is required for infection. A549 cells were pre-treated with non-cytotoxic concentrations of the indicated Src-kinase inhibitors (PP2 [12.5 μM], SrcI-I [6.25 μM] or both) or mock-treated (DMSO carrier control) for 5 h. Cells were then inoculated with RSV-GFP (MOI = 1 PFU/cell) in media containing the indicated inhibitors. **(A) Effect of Src-kinase inhibition on RSV infection.** At 17 h p.i., GFP intensity was measured with an ELISA reader and expressed relative to mock-treated infected cells assigned the value of 1.0. **(B) Titer of progeny RSV-GFP.** At 24 h p.i. RSV titers were determined in replicate cultures by plaque titration on Vero cells. The statistical significance of the difference was determined by one-way analysis of variance with Tukey’s multiple-comparison post-test and p-values are shown for each comparison.

We next investigated the downstream signaling pathways of ATP1A1 that might be involved in ATP1A1-dependent RSV entry. Binding of ouabain to ATP1A1 activates the c-Src-kinase that transactivates EGFR signaling [17]. Therefore, we investigated whether c-Src activity is needed for RSV infection, which was done using two Src-kinase inhibitors PP2 and Src-Inhibitior I (SrcI-I). A549 cells were pre-treated with these inhibitors separately or together for 5 h, using concentrations that preliminary studies showed were non-cytotoxic (S4 Fig), followed by infection with RSV-GFP (MOI = 1 PFU/cell) in the continued presence of inhibitors. The efficiency of RSV infection was evaluated by (i) GFP expression at 17 h p.i. for all treatments (Fig 6A), and (ii) RSV titration at 24 h p.i. for cells that had been treated with both inhibitors (SrcI-I+PP2) (Fig 6B). Each Src-inhibitor showed a modest but significant (p < 0.0001) reduction in GFP intensity of 23% (SrcI-I) and 33% (PP2) relative to mock-treated infected cells. When added together, the inhibitory effect increased to 45% reduction compared to mock-treated infected cells. The RSV titer (PFU) for the combined Src-inhibitor treatment showed a 2-fold, significant (two-tailed, unpaired t-test, p = 0.0014) reduction compared to mock-treated infected cells. These data confirmed that Src-kinase activity contributes to RSV infection.

### EGFR knockdown reduces RSV infection

**Fig 7:**
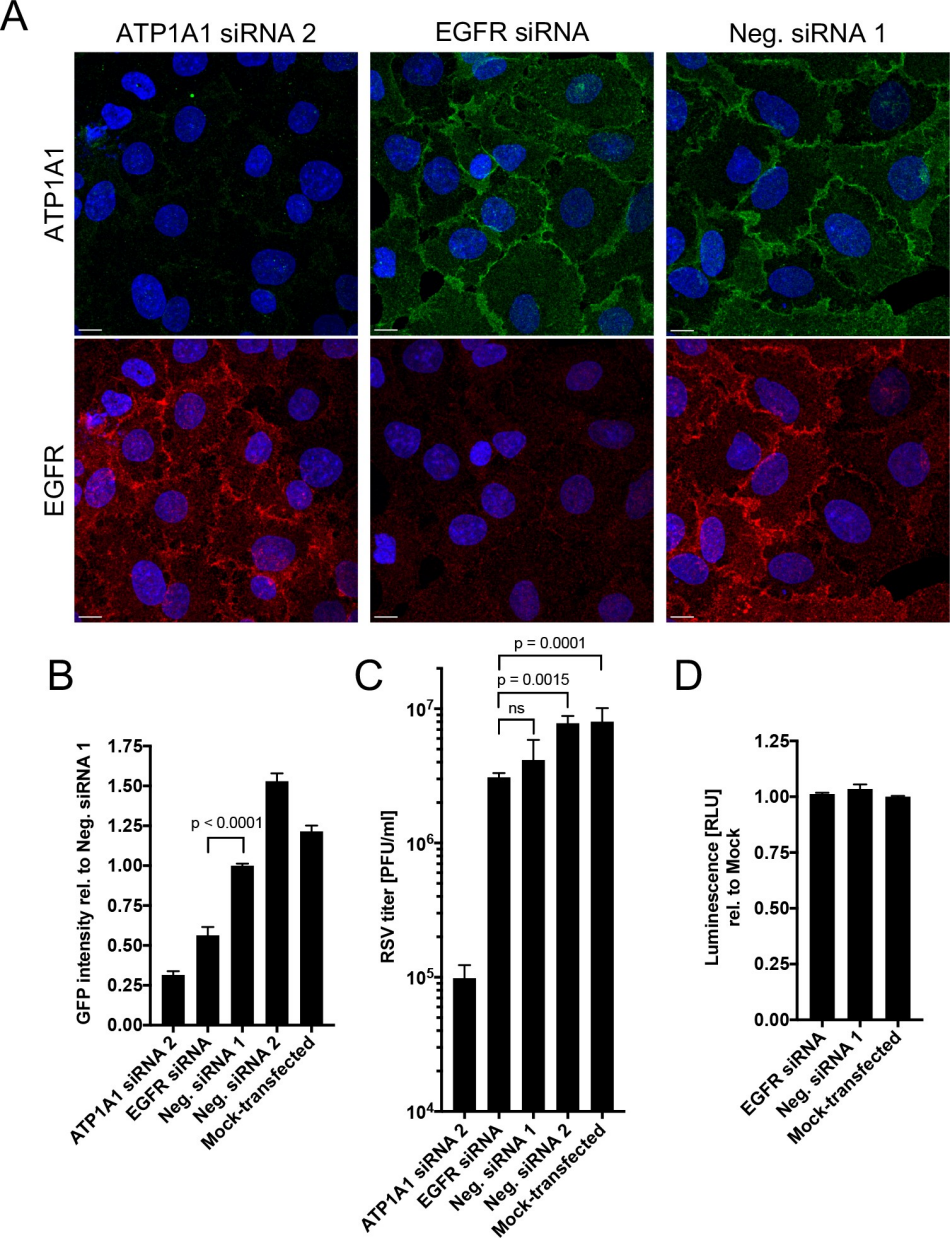
Effect of EGFR knockdown on RSV infection. **(A) ATP1A1 and EGFR expression in A549 cells transfected with siRNAs targeting ATP1A1 or EGFR.** A549 cells were transfected with ATP1A1 siRNA 2, EGFR siRNA, or Neg. siRNA 1 or 2, incubated for 48 h, and subjected to immunofluorescence staining for ATP1A1 (green) and EGFR (red) as described for Fig 4A. Images (z-stacks) were acquired on a Leica SP5 confocal microscope with 63x objective NA 1.4 and 2.0x zoom. Scale bar 10 μm. **(B and C) EGFR knockdown reduces RSV infection and virion production.** A549 cells were transfected with an EGFR-specific siRNA or Neg. siRNA 1 or 2, and 48 h later were infected with RSV-GFP (MOI = 1 PFU/cell). Infection was quantified by (B) GFP signal of the total well, scanned by ELISA reader at 17 h p.i., and RSV production was assessed by (C) plaque titration on Vero cells 24 h p.i. Data are derived from three independent experiments. The statistical significance of the difference was determined by one-way analysis of variance with Tukey’s multiple-comparison post-test and p-values of the significance for each comparison is indicated. **(D) Cell viability.** An ATP-based cell viability assay (CellTiter-Glo) was performed 72 h p.t. to evaluate the viability of the transfected cells. Cells were lysed, the ATP concentration was determined by luciferase activity relative to mock-transfected cells, as a measure of viability.

Next, we investigated whether EGFR, a downstream signaling partner of Src-kinase, made a contribution to RSV infection. We identified an EGFR-specific siRNA that reduced EGFR expression in A549 cells to 15% at the protein level compared to Neg. siRNA1 48 h p.t. (S5 Fig), with minimal effect on cell viability. A549 cells were transfected with EGFR-specific, ATP1A1-specific, or control siRNA for 48 h and evaluated by immunofluorescence staining for EGFR and ATP1A1. This showed that the ATP1A1 and EGFR siRNAs greatly reduced the expression of their corresponding target proteins (Fig 7A, ATP1A1 top panel; EGFR: bottom panel) without affecting EGFR and ATP1A1, respectively, whose expression remained similar to that of Neg siRNA 1.

EGFR knockdown cells were infected with RSV-GFP and infection was evaluated by viral GFP intensity quantified by ELISA reader 17 h p.i. (Fig 7B) and by plaque titration 24 h p.i. on Vero cells (Fig 7C). EGFR knockdown resulted in a nearly 50% reduction in viral GFP expression (Fig 7B) as compared to Neg. siRNA 1 (p = 0.0001). There also was a 38% reduction in RSV titer compared to Neg. siRNA 2 (p = 0.0015) or mock-transfected cells (p = 0.0001) (Fig 7C). There was a modest but consistent reduction in PFU titer for Neg. siRNA1 for unknown reason and hence the reduction in titer for EGFR siRNA treated cells was not significantly lower compared to this particular control siRNA (ns, p = 0.9891), but as noted above the reduction in GFP expression was highly significant. None of the siRNAs had an effect on cell viability (Fig 7D). Taken together, these data confirm that EGFR plays a role in RSV infection.

### EGFR Tyrosine 845 is phosphorylated in response to RSV infection and is ATP1A1 dependent

**Fig 8:**
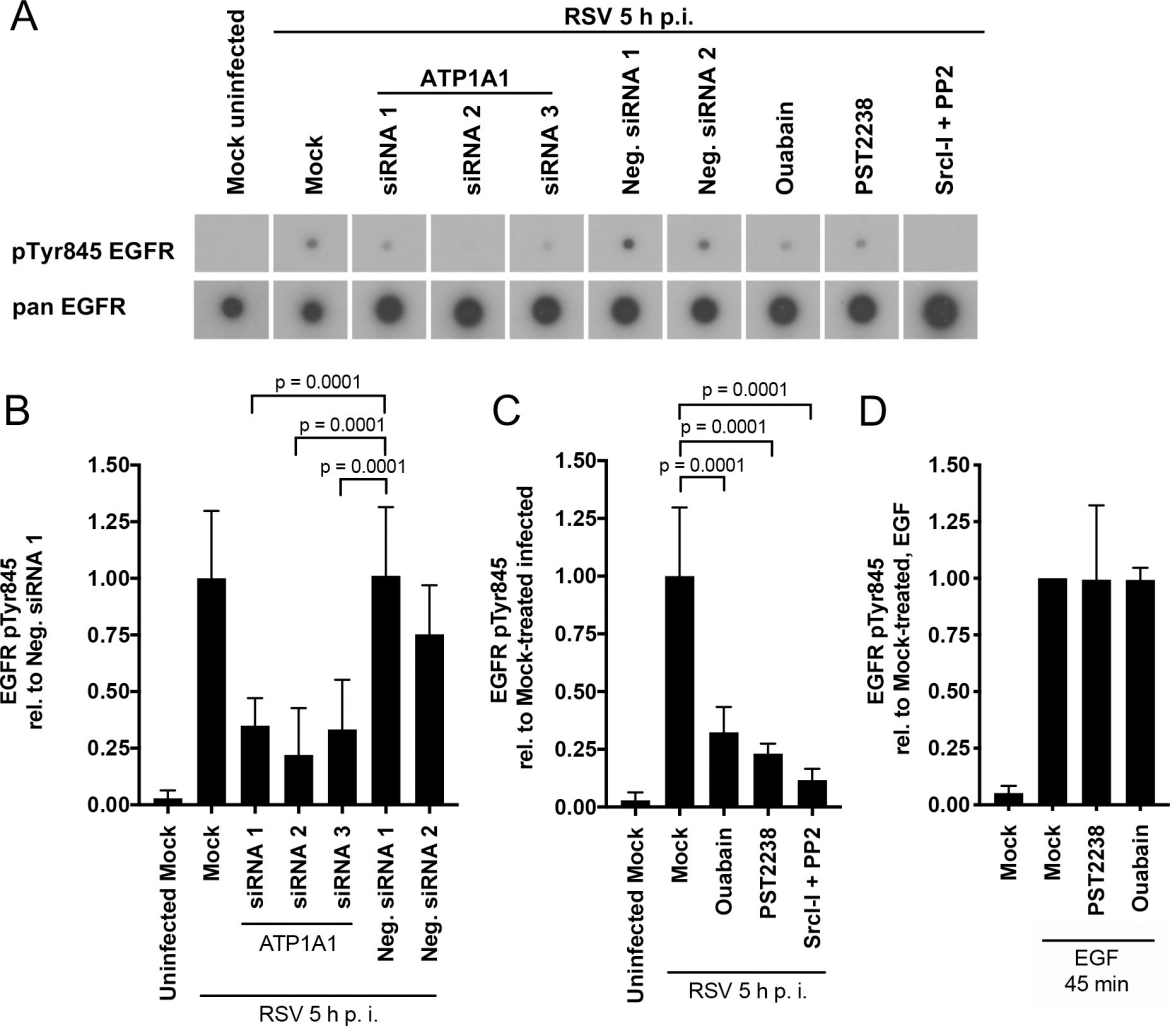
ATP1A1-dependent EGFR phosphorylation at Tyr845 during RSV infection. A549 cells were treated as indicated (either siRNA knockdown for 48 h, or pre-treatment with the chemical compounds ouabain [25 nM], PST2238 [40 μM] or Src-Inhibitor-I (SrcI-I) [6.25 μM] and PP2 [12.5 μM] for 5 h pre-inoculation). Coincident with these treatments, the cells were serum starved for 16 h, and then were inoculated with wt RSV (MOI = 5 PFU/cell) and incubated for 5 h. The cells were lysed and lysates were incubated with a phospho-specific EGFR antibody array, followed by detection of bound EGFR with a biotinylated pan (i.e., universal) EGFR antibody, followed by incubation with horse radish peroxidase-conjugated streptavidin, and visualization with X-ray film (RayBiotech, Inc.). **(A)** X-ray film showing representative array spots of pTyr845 EGFR and its corresponding pan EGFR for each treatment. **(B—D) Quantification of EGFR Tyr845 phosphorylation.** pTyr845 EGFR signals of three independent experiments with two technical replicates each were quantified by scanning and normalized to the signal of the internal array positive controls and pan EGFR. (B) shows the siRNA knockdown samples reported relative to Neg. siRNA 1-transfected, RSV-infected samples, and (C) shows the chemical-treated samples reported relative to mock-treated, RSV-infected samples. (D) As control, PST2238 or Ouabain pretreated A549 cells were stimulated with EGF (100 ng/ml) for 45 min and the pTyr845 EGFR signal was quantified. The statistical significance of the differences were determined by one-way analysis of variance with Dunnett multiple-comparison test and the p-values are indicated for each comparison.

We next investigated EGFR phosphorylation during RSV infection. As described in Fig 8, A549 cells were pretreated by transfection with ATP1A1 siRNA 1–3, or Neg. siRNAs 1 and 2, or were pretreated with ouabain, PST2238, or the Src-inhibitors SrcI-1+PP2. The cells were then infected with RSV (MOI = 5 PFU/cell) and lysates were prepared following 5 h of incubation. The lysates were analyzed using an EGFR phosphorylation array to identify the EGFR sites that were phosphorylated during infection.

EGFR in RSV-infected A549 cells was phosphorylated at Tyr845, which was not detected for uninfected cells (Fig 8 and S6A Fig), whereas both samples showed nearly equivalent phosphorylation of ErbB2 (another member of the human EGFR family) at Thr686 and Ser1113 (S6 Fig). The level of pTyr845 in RSV-infected cells was significantly (p < 0.0001) reduced in the ATP1A1 siRNA knockdown cells, to an average of 35% (siRNA1), 22% (siRNA2), and 33% (siRNA3) relative to Neg. siRNA1 (Fig 8B). The phosphorylation of Tyr845 in RSV-infected cells was similar to Neg. siRNA1. While it was slightly reduced for Neg. siRNA2, the difference was not significant (p = 0.3651) compared to Neg. siRNA1. Consistent with the ATP1A1 knockdown, a significant reduction in Tyr845 phosphorylation also was observed when the cells were pre-treated with ouabain, PST2238, or Src-kinase inhibitors (SrcI-I +PP2) prior to infection with RSV (Fig 8C). For ouabain- and PST2238-treated cells, the level of pTyr845 was reduced to 27% and 26%, respectively, compared to mock-treated RSV-infected cells; the reduction was similar to that observed for ATP1A1 knockdown. To confirm a lack of a direct inhibitory effect of Ouabain or PST2238 on EGFR, A549 cells were pre-treated with PST2238 or Ouabain and stimulated with EGF for 45 min. The EGF-induced phosphorylation of pTyr845 indeed was not affected by the compounds as compared to mock-treated cells (Fig 8D). The Src-kinase inhibitors (SrcI-I+PP2) reduced phosphorylation at Tyr845 to 12% compared to mock-treated infected cells (Fig 8C). Thus, phosphorylation at EGFR Tyr845 could be reduced either by decreasing ATP1A1 expression or by ATP1A1- or Src-specific inhibitors. This suggested that EGFR pTyr845 is ATP1A1-dependent and that Src-kinase serves as a signaling effector to transactivate EGFR by Tyr845 phosphorylation.

### Macropinocytosis is induced by RSV and is mediated by ATP1A1

**Fig 9:**
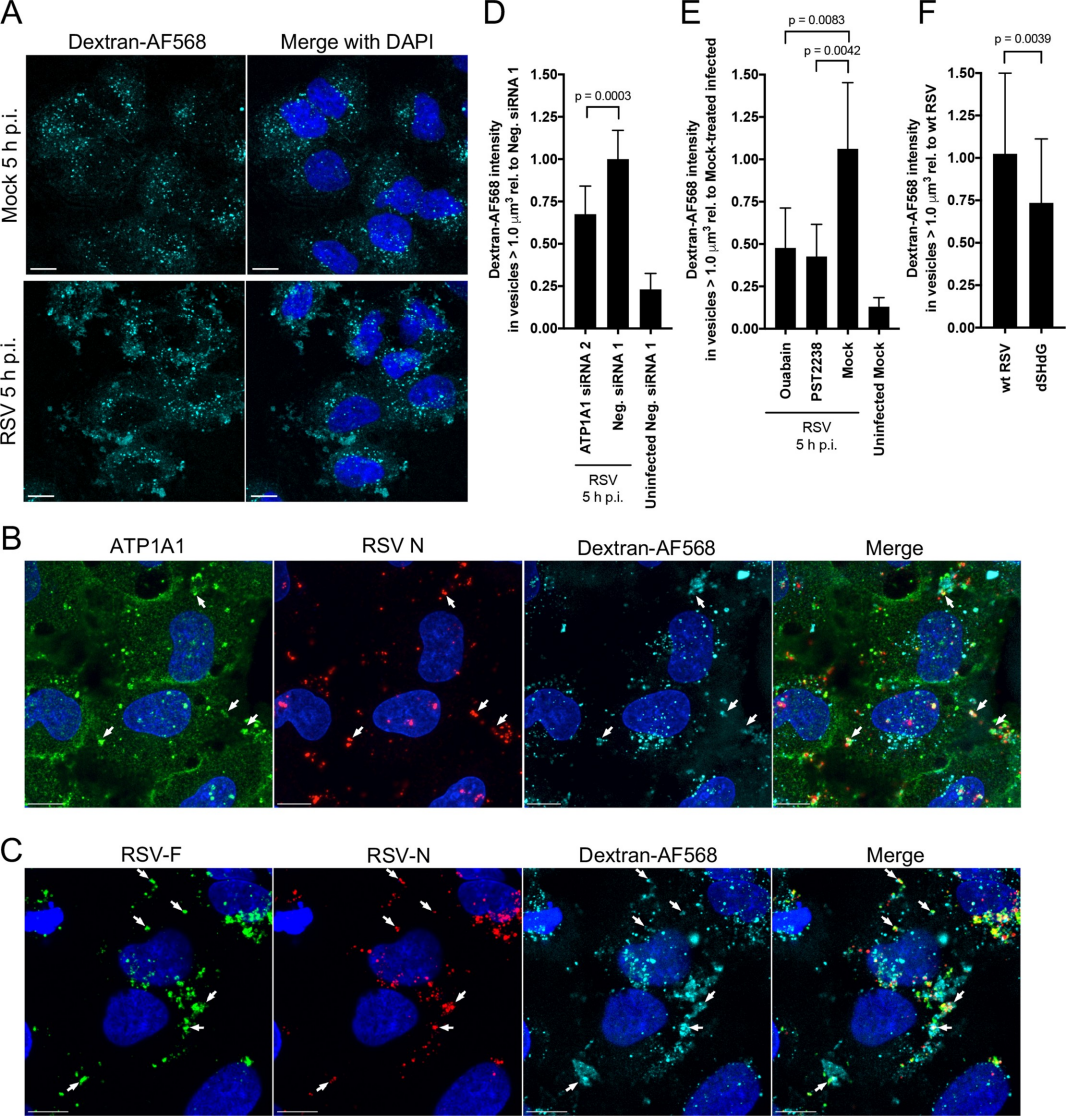
RSV induces and is taken up by ATP1A1-dependent macropinocytosis, which can be blocked by ouabain or PST2238. Macropinocytosis was assayed by monitoring the uptake of dextran (10,000 MW) conjugated to AF568 (dextran-AF568). All incubations with dextran-AF568 were preceded by serum-starvation for 16 h. **(A) RSV induces macropinocytosis.** A549 cells were mock-infected or infected with wt RSV (MOI = 5 PFU/cell) in medium containing dextran-AF568 (cyan). At 5 h p.i., cells were fixed with 4% PFA and nuclei counterstained with DAPI (blue), and imaged on a Leica SP5 confocal microscope with a 40x Objective NA 1.3 and 2.0x zoom. **(B) Co-localization of ATP1A1, RSV N, and dextran-AF568 in RSV-infected A549 cells**. Cells were infected with RSV in the presence of dextran-AF568 as described above, incubated for 5 h, fixed with 4% PFA, permeabilized with 0.1% Triton X-100, subjected to immunofluorescence staining with an anti-ATP1A1 rabbit MAb (ab76020) and an anti-RSV-N mouse MAb (ab94806), followed by AF488-conjugated goat anti-rabbit and AF647-conjugated goat anti-mouse secondary antibodies. Z-stacks were acquired on Leica SP8 confocal microscope with 63x objective, NA 1.4 and 3.0x zoom. Arrows indicate co-localization of ATP1A1 (green) and RSV N (red) in dextran-AF568-positive (cyan) vesicles. **(C) Co-localization of RSV F and RSV N with dextran-AF568 in RSV-infected A549 cells.** Cells were infected with RSV in the presence of dextran-AF568, incubated for 5 h, fixed, and permeabilized as described in B. The cells were then subjected to immunostaining: RSV F was detected with AF488-conjugated anti-RSV F MAb #1129 [72], and RSV N was detected with an allophycocyanin (APC)-conjugated anti-RSV N MAb (NB100-64752APC, Novus Biologicals, LLC). Image acquisition and analysis were performed as described above for B. Arrows indicate RSV F (green) and RSV N (red) in dextran-AF568-positive (cyan) vesicles. All scale bars are 10 μm. **(D–F) Quantification of dextran-AF568 uptake during RSV infection.** (D) A549 cells were transfected with ATP1A1 siRNA2 or Neg. siRNA 1, incubated for 48 h p.t., and inoculated with wt RSV in dextran-AF568-containing medium, or (E**)** A549 cells were pre-treated with ouabain or PST2238 for 16 h and inoculated with wt RSV in dextran-AF568-containing medium, or (F**)** A549 cells were infected with wt RSV or rgRSV dSH/dG in dextran-AF568-containing medium. For all treatments (D-F) cells were fixed 5 h p.i., counterstained with DAPI and z-stacks were acquired on a Leica SP8 confocal microscope with 63x objective NA 1.4, 1.0x zoom. For each treatment, the uptake of dextran-AF568 in vesicles greater than 1.0 μm^3^ was quantified as described in detail in the Materials and Methods section. Mean values are reported relative to RSV-infected cells transfected with Neg. siRNA 1 (D), or mock-treated infected cells (E), or wt RSV-infected cells (F). Error bars indicate the standard deviation of at least three independent experiments. The statistical significance of difference was determined for (D) and (E) by one-way analysis of variance with Tukey’s multiple comparison post-test and for (F) by a two-tailed unpaired t-test. P-values are shown for each comparison.

EGFR signaling is known to cause actin rearrangement, membrane ruffling, and activation of endocytosis and macropinocytosis [31, 33, 34]. Macropinocytosis is a nonspecific, fluidic uptake at the cell surface that initiates through actin rearrangement and membrane ruffling that give rise to large vesicles called macropinosomes. Limited prior evidence suggested macropinocytosis as a route of RSV uptake [14, 16]. In the present study, A549 cells were infected with wt RSV (MOI = 5 PFU/cell) in the presence of dextran (10,000 MW) conjugated to fluorescent dye (Alexa Fluor [AF] 568) as a fluidic uptake marker. At different time points p.i., cells were washed and fixed, nuclei were counterstained with DAPI, and the uptake of dextran was analyzed by fluorescence confocal microscopy. Cells that had been mock-infected were found to contain dextran-positive vesicles that were small, round, and homogeneous in size with an average volume of ~ 0.5 μm^3^ (Fig 9A, top panel), reflecting the basal level of dextran uptake. This phenotype changed dramatically by 5 h after infection with wt RSV (Fig 9A, bottom panel). The dextran-positive vesicles were much bigger (average volume of ~5.7 μm^3^) and irregular in shape, as is typical of macropinosomes. The induction of large dextran-positive vesicles also was visible at earlier time points and could be detected as early as 30 min p.i. and became more prominent at 1 h p.i. (S7 Fig). This showed that RSV infection induces macropinocytosis.

Next, A549 cells were infected with RSV in the presence of dextran-AF568 (cyan), and at 5 h p.i. were co-stained for ATP1A1 (AF488; green) and RSV N (marker of RSV virions; AF647; red) and visualized by fluorescence confocal microscopy. As previously noted, 5 h p.i. is very early in RSV infection, and the N protein that is detected would be mainly from the input virus particles, as was shown earlier with UV-inactivated virus. Clusters of ATP1A1 were observed co-localized with RSV N protein in the dextran-positive macropinosomes (Fig 9B, indicated by arrows), indicating that RSV was indeed taken up by macropinocytosis. Co-staining for RSV F and N was also performed and showed that both proteins were co-localized in the dextran-positive macropinosomes (Fig 9C). The presence of RSV F suggests that the RSV detected in the macropinosomes was enveloped, indicating that fusion and release of nucleocapsid presumably occurs subsequently in the internal vesicles rather than at the plasma membrane.

To examine the role of ATP1A1 in this putative uptake mechanism, ATP1A1 expression was knocked down with siRNA, or the cells were treated with ouabain or PST2238 as previously described. Next, these cells were infected with RSV (MOI = 5 PFU/cell) in the presence of dextran-AF568, followed by fluorescence confocal microscopy. Multiple random Z-stack images were acquired by confocal microscopy, and dextran-positive vesicles were analyzed by Imaris imaging software to determine the total fluorescence intensity per vesicle. Vesicles smaller than 1.0 μm^3^ were excluded to omit the basal level of dextran uptake and to focus on the large vesicles that are typical for macropinosomes. The total intensity of dextran vesicles larger than 1.0 μm^3^ was determined per field, normalized to the number of nuclei and expressed relative to Neg. siRNA1-transfected cells (Fig 9D). This showed that dextran uptake was increased 4-fold in RSV-infected as compared to uninfected-cells, which had both been transfected with Neg. siRNA1 (Fig 9D), confirming the visual observation of increased dextran-AF568 uptake (Fig 9A). On the other hand, knockdown of ATP1A1 caused a significant (p = 0.0003) reduction of 33% compared to Neg. siRNA1 (Fig 9D). Ouabain and PST2238 caused an even-greater reduction in RSV-induced macropinosomes, to less than 50% compared to mock-treated, RSV-infected cells (Fig 9E). Since RSV G was suggested to be important for triggering ATP1A1 activation, based on the loss of clustering observed with the dSH/dG mutant as described previously in Fig 4, we also quantified macropinosomes in cells infected with wt RSV or the dSH/dG RSV mutant. This showed that macropinosome formation indeed was significantly (p = 0.0039) reduced for the dSH/dG virus as compared to wt RSV (Fig 9F), consistent with a role for the G protein in activating the pathway leading to macropinosome formation.

Signaling from ATP1A1 also can induce clathrin-mediated endocytosis [17, 26], and this endocytic pathway has been controversially suggested to be involved in RSV uptake [15]. However, preliminary studies in our hands using an inhibitor of clathrin-mediated endocytosis (e.g., chlorpromazine) did not detect effects on RSV infection at non-cytotoxic concentrations (S8 Fig), and this was not pursued further.

### Cholesterol is required for RSV uptake and signaling

ATP1A1-Src-EGFR signaling characteristically is associated with caveolae [17, 26–28]. The structural integrity of caveolae depends on the presence of cholesterol, and cholesterol-depletion disrupts caveolae from the plasma membrane [39, 40]. We therefore evaluated the impact of depleting cholesterol in A549 cells prior to RSV infection, which was done using methyl-beta-cyclodextrin (MBCD) and Mevinolin, individually or in combination at non-cytotoxic concentrations (S4 Fig). MBCD removes cholesterol from the plasma membrane whereas Mevinolin inhibits its biosynthesis.

**Fig 10:**
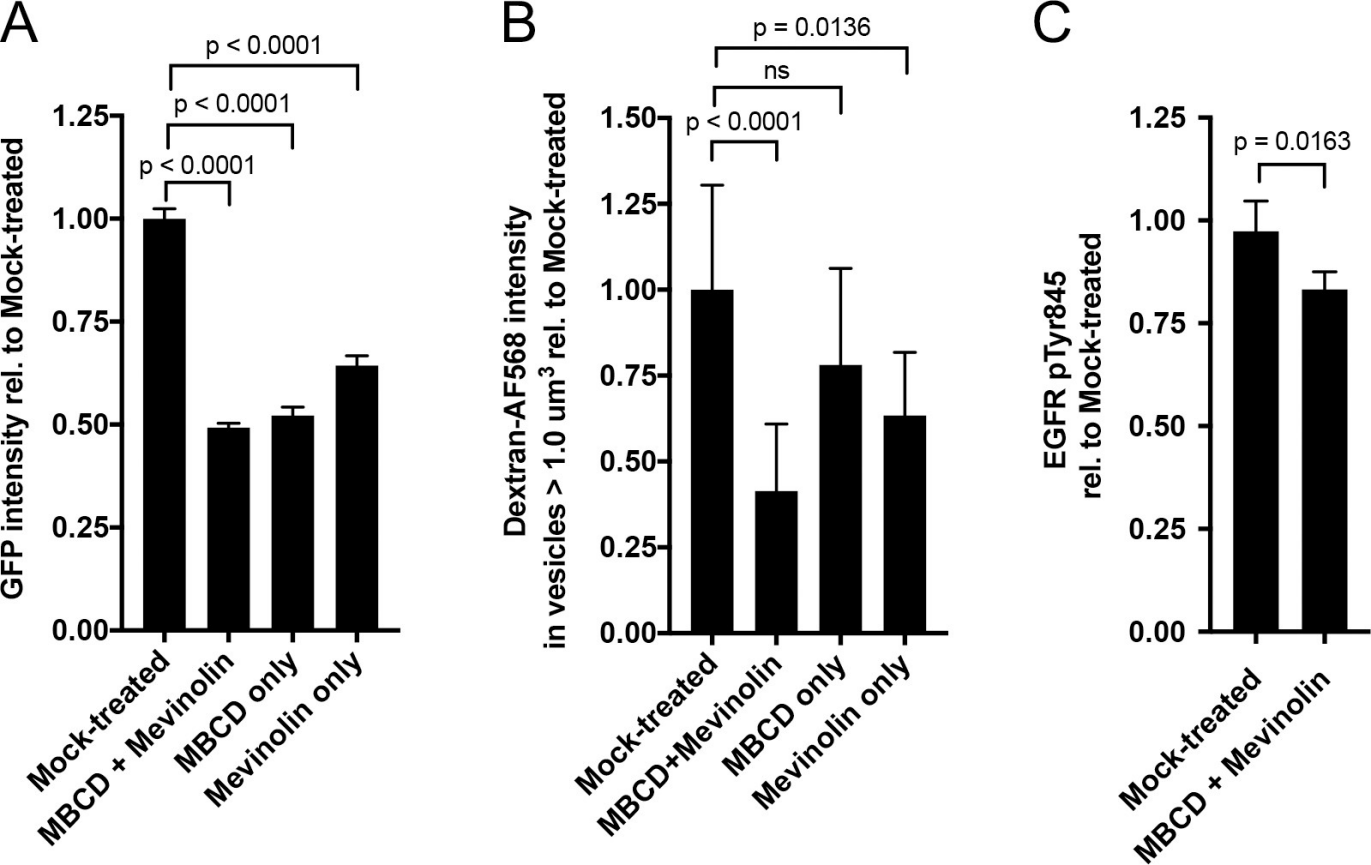
Effect of cholesterol depletion on RSV infection. A549 cells were cholesterol-depleted by treatment with methyl-beta-cyclodextrin (MBCD) and Mevinolin or each chemical separately. **(A) RSV infection of cholesterol-depleted A549 cells.** A549 cells were pre-treated for 5 h with the indicated cholesterol-depleting compounds and infected with RSV-GFP (MOI = 1 PFU/cell) with presence of the cholesterol-depleting compounds maintained. Viral GFP expression was quantified 17 h p.i. and reported relative to mock-treated infected cells. **(B) Quantification of macropinocytosis in cholesterol-depleted RSV-infected A549 cells.** A549 cells were pre-treated for 16 h with the indicated cholesterol-depleting compounds in the absence of serum and infected with RSV (MOI = 5 PFU/cell) in the presence of dextran-AF568. At 5 h p.i the cells were fixed with 5% PFA and nuclei were counterstained with DAPI. The total intensity of dextran-AF568 in vesicles larger than 1.0 μm^3^ was quantified (Materials and Methods) and reported relative to mock-treated infected cells. The statistical significance of the differences was determined by one-way analysis of variance with Tukey’s multiple-comparison post-test and p-values are indicated (ns, not significant)**. (C) EGFR Tyr845 phosphorylation in cholesterol-depleted cells.** A549 cells were treated with MBCD and Mevinolin for 16 h to deplete cholesterol from the plasma membrane. Cells were infected with wt RSV (MOI = 5 PFU/cell) and the phosphorylation of EGFR Tyr845 was quantified by an EGFR phosphorylation antibody array, as described in Fig 8. The level of pTyr845 was reported relative to mock-treated infected cells. The statistical significance of difference was determined by a two-tailed unpaired t-test.

First, we infected cholesterol-depleted A549 cells with RSV-GFP and found that GFP expression at 17 h p.i. was reduced to approximately 50% with each of the depletions, compared to control infected cells (Fig 10A). Next, we quantified the level of RSV-induced macropinocytosis in cholesterol-depleted cells by dextran-AF568 uptake into large vesicles. In RSV-infected cells, dextran uptake was reduced by each of the cholesterol-depletion treatments, with the most significant (p < 0.0001) reduction of 59% observed for the combined MBCD-Mevinolin treatment (Fig 10B). The phosphorylation of EGFR Tyr845 also was determined in RSV-infected cells pretreated with MBCD + Mevinolin. A modest but significant (two tailed t-test, p = 0.0163) reduction in pTyr845 was observed (Fig 10C). These results show that cholesterol depletion results in reduced EGFR transactivation, reduced macropinocytosis, and reduced RSV infection, consistent with caveolae as the site of ATP1A1 signaling.

### Validation of findings in primary human small airway epithelial cells and differentiated human airway epithelial air-liquid interface (HAE-ALI) cultures

**Fig 11:**
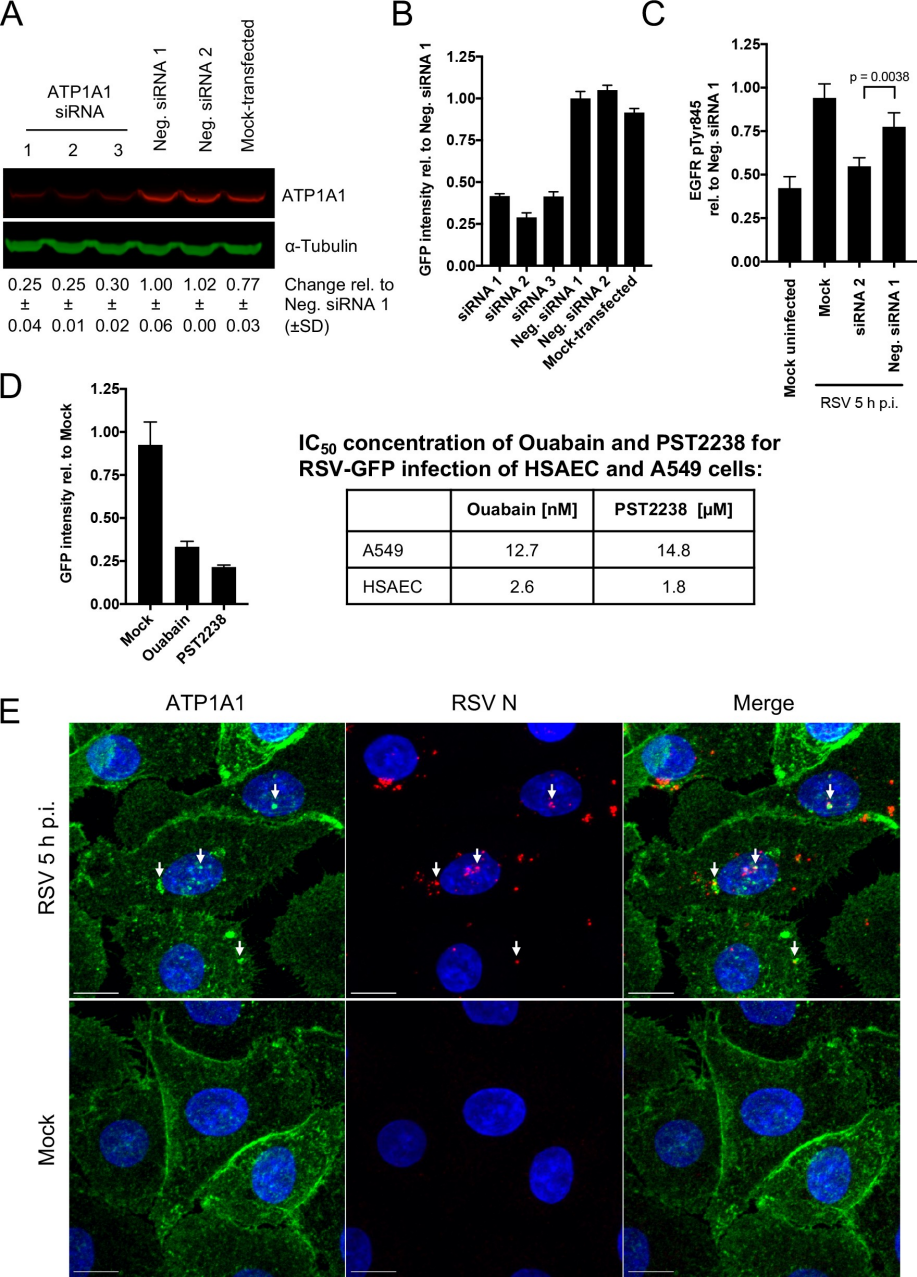
Confirmation of results obtained in the A549 cell line by experiments in primary human small airway epithelial cells (HSAEC). Experiments were performed as described for A549 cells in the Materials and Methods section and the preceding Figure legends. **(A)** siRNA knockdown in HSAEC using ATP1A1 siRNAs 1, 2, and 3 and Neg. siRNAs 1 and 2, analyzed 48 h p.t., by Western blot analysis as in Fig 1B. **(B)** Efficiency of RSV infection in HSAEC cells following knockdown of ATP1A1 expression using the indicated siRNAs, assayed by GFP quantification by ELISA reader, as in Fig 2A. **(C)** EGFR Tyr845 phosphorylation in HSAEC cells following knockdown of ATP1A1 expression using ATP1A1 siRNA 2, as in Fig 8. **(D)** Inhibition of RSV-GFP infection in HSAEC cells treated with 3.1 nM ouabain or 3.1 μM PST2238, evaluated by the expression of GFP as in Fig 5B. The IC_50_ values of ouabain and PST2238 for inhibiting RSV infection were 5- and 8-fold lower than for A549 cells, respectively (S3A and S3B Fig). **(E)** ATP1A1 clustering and colocalization with RSV N protein in HSAEC cells, in an experiment performed as in Fig 3. Scale bar 10 μm.

All experiments described above were performed with the human airway epithelial A549 cell line. We sought to confirm some of the major findings using primary human small airway epithelial cells (HSAEC) from a 16-year old healthy male donor. SiRNA transfection knocked down the expression of ATP1A1 protein to 25–30% compared to Neg. siRNA 1 (Fig 11A), which was somewhat more than the reduction to 35–39% observed for A549 cells. Following the same protocols as for A549 cells, we found that (i) knockdown of ATP1A1 reduced the expression of RSV-GFP to 29–42% of the negative control (Fig 11B), similar to what was observed with A549 cells (Fig 2A). (ii) Phosphorylation of EGFR Tyr845 was also significantly (p = 0.0038) reduced in ATP1A1-knockdown (siRNA2) HSAEC (Fig 11C), similar to what was seen in A549 cells (Fig 8). (iii) The inhibitory effects of ouabain and PST2238 on the expression of RSV-GFP were even stronger in HSAEC than in A549 cells (Fig 11D). IC_50_ titrations of the compounds on HSAEC for RSV-GFP expression showed that the values were lower (i.e., more effective inhibition) in HSAEC than A549 cells by 4.9-fold for ouabain (S3A Fig) and 8.2-fold for PST2238 (S3B Fig). (iv) RSV-induced ATP1A1 clustering and colocalization of ATP1A1 and RSV N also was observed in HSAEC (Fig 11E), similar to A549 cells (Fig 3).

**Fig 12:**
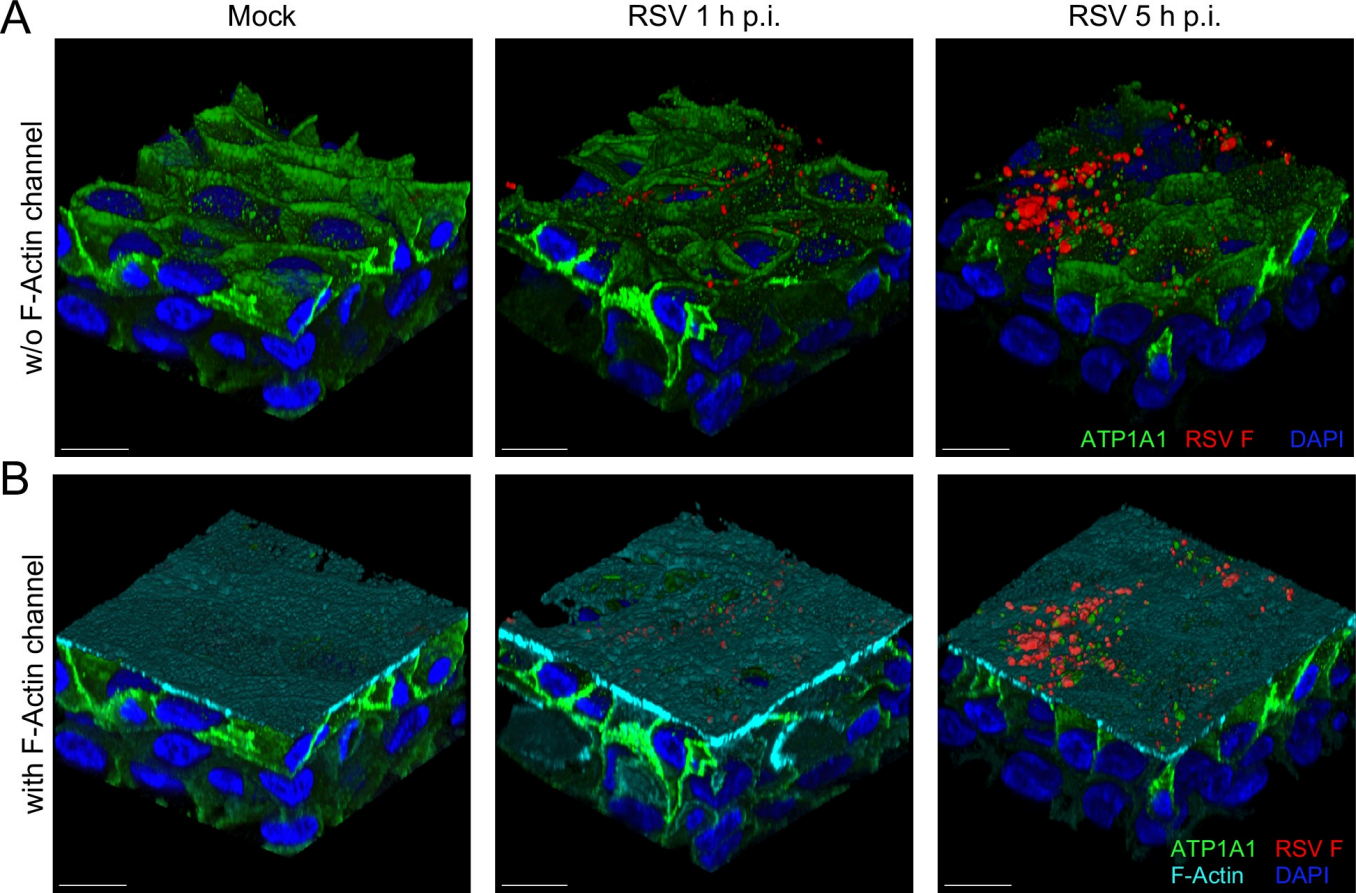
RSV induces ATP1A1 clustering in primary human airway epithelial-air liquid interface (HAE-ALI) cultures. HAE-ALI cultures were inoculated with wt RSV (10^6^ PFU/tissue), incubated for 1 or 5 h, fixed and subjected to immunofluorescence staining as described for Fig 3 (ATP1A1, green; RSV F, red; F-Actin, cyan; DAPI; blue). Images are shown without (A) and with (B) F-Actin staining. Images were deconvolved with Huygens Essential deconvolution software (Scientific Volume Imaging B.V, Hilversum, The Netherlands). Scale bars 10 μm.

In addition, we used HAE-ALI cultures, a model of primary, differentiated, polarized mucociliary airway epithelium, to investigate the localization of ATP1A1 and to confirm the phenomenon of RSV-induced ATP1A1 clustering. Cells were infected with wt RSV (10^6^ PFU/transwell), incubated for 1h or 5 h, fixed with PFA, permeabilized with TritonX-100, and immunostained for RSV F (red) and ATP1A1 (green), as described for A549 cells in Fig 3. The apical surface was demarcated by staining for F-actin (cyan) since it is abundant beneath the apical membrane and provides a close estimation of the apical surface location. To visualize ATP1A1 location, three-dimensional sections are shown without (Fig 12A) and with (Fig 12B) F-actin staining. In mock treated cells, ATP1A1 was predominantly present in the basolateral surfaces of cells with relatively smaller amounts present on the apical surface in a spotted distribution, (Fig 12, left panel). Upon infection with RSV, ATP1A1 clusters were visible as early as 1 h p.i. (Fig 12A and 12B, middle panel), which became more noticeable and larger at 5 h p.i. (Fig 12A and 12B right panel). The apical ATP1A1 seemed to increase in amount over time following infection, suggesting its recruitment to the apical surface. The ATP1A1 clusters were mostly visible in close proximity to RSV F. These observations were similar to those in A549 cells (Fig 3), confirming that RSV-induced ATP1A1 clustering could be reproducibly demonstrated in two different primary cell systems.

## Discussion

In the present study, we investigated the host proteins involved in RSV infection using a genome-wide high-throughput siRNA screen in human airway A549 cells infected with a recombinant RSV that expresses GFP. Knockdown of the cellular ATP1A1 protein provided one of the greatest reductions in GFP expression with minimal effects on cell viability. ATP1A1 is the major subunit of Na^+^,K^+^-ATPase, a transmembrane complex that is an ATPase, an ion channel, and also is involved in signal transduction [17]. In this study, we provided evidence that ATP1A1—in particular its signal transduction function—is needed for efficient RSV infection.

We attempted to evaluate the effect of reduced ATP1A1 expression on RSV infection *in vivo* using a heterozygous ATP1A1 knockdown mouse strain (B6;129S5-Atp1a1^Gt(neo)311Lex/Mmucd^; from the Mutant Mouse Resource & Research Centers (MMRRC), University of California, Davis, RRID: MMRRC_011687-UCD). It was necessary to use the heterozygous ATP1A1^+/-^ genotype because the homozygous ATP1A1^-/-^ knock-out is lethal at the preweaning stage. No significant difference in the virus load was observed between ATP1A1^+/-^ mice and their wild-type litter mates. This was not surprising, given the modest reduction in ATP1A1 expression (reduced only by 30–35% compared to wt litter mates), which presumably was not sufficient to reduce the efficiency of RSV infection.

Two ATP1A1-binding drugs, ouabain and PST2238, were found to inhibit RSV infection. Inhibition of RSV infection with ouabain was achieved at sub-nanomolar concentrations that initiate ATP1A1 signaling cascades [17, 26] but are not inhibitory for its ATPase and ion channel functions and do not alter the cytosolic Na^+^ and K^+^ levels [41]. While incubation with ouabain depletes Na^+^,K^+^-ATPase over time from the plasma membrane, it is unlikely that this depletion was the sole mechanism for the inhibition of RSV infection, because the antiviral effect of ouabain was evident even when added simultaneously with the virus inoculum. We speculate that the mechanism of inhibition by shorter-term treatment with ouabain involves competition with RSV for ATP1A1 signaling, as is discussed later. Treatment with PST2238 also reduced the efficiency of RSV infection. PST2238 is a competitive inhibitor of ouabain that shares a common binding site on the extracellular domain of ATP1A1. PST2238 does not induce signaling and indeed blocks ATP1A1 signaling in response to ouabain or RSV. Both of these ATP1A1-binding drugs implicated ATP1A1—and in particular ATP1A1 signaling—as being important for efficient RSV infection. Time-of-addition experiments indicated that the inhibitory effects of ouabain and PST2238 occurred very early during infection.

We observed a striking phenomenon in which ATP1A1 formed clusters in the plasma membrane within 15 minutes following infection with RSV. This clustering was not affected by treatment with PST2238, indicating that clustering does not depend on ATP1A1 signaling. Clustering of ATP1A1 occurred with equal efficiency with UV-inactivated RSV, and thus was independent of transcription of the complete genome, viral RNA replication, and virus replication. This clustering was reminiscent of the behavior of signaling receptors following ligand binding, and suggested there might be a physical interaction between the virions and the cell surface that triggers ATP1A1 clustering. Interaction of this sort could be imagined as part of viral attachment or part of a cellular response to RSV virions. However, we were unable to detect binding between ATP1A1 and any of the RSV surface glycoproteins (G, F or SH) by various co-immunoprecipitation techniques. For example, we investigated possible binding using the human lung epithelial cell line H1299 ATP1A1-YFP (kindly provided by Uri Alon, Weizmann Institute of Science, Rehovot, Israel [42, 43]) that chromosomally expressed ATP1A1 genetically fused to yellow fluorescent protein (YFP) tag. These cells were infected with RSV, incubated for 1h and lysed, and co-immunoprecipitation was performed with YFP-specific antibodies followed by Western blotting with antibodies to RSV proteins. However, there was no evidence of pull-down of RSV proteins. We also performed comparable co-precipitation experiments in which, prior to lysis, cells were treated with the permeable, reversible cross-linker dithiobis (succinimidyl propionate)[44]. We also incubated RSV virions with ATP1A1 immobilized on beads. However, there was no evidence of binding between ATP1A1 and RSV proteins in these experiments. We also performed a cell-based binding assay [45], using A549 cells that had been siRNA-transfected to knock down ATP1A1. Specifically, the cells were transfected with ATP1A1 siRNA, incubated for 48 h, detached, and incubated on ice with RSV for 30 min, after which the cells were extensively washed and incubated with a pool of RSV F-specific monoclonal antibodies to detect possible bound virus. ATP1A1 knockdown did not result in an appreciable reduction in RSV binding, and thus we did not detect any contribution of ATP1A1 to RSV attachment. Treatment of the cells with heparinase I prior to RSV exposure to remove cell surface GAGs, which was done to eliminate GAG-mediated RSV attachment, also did not reveal any contribution of ATP1A1 to RSV attachment. Therefore, there was no evidence of stable binding of any RSV surface protein to ATP1A1.

It may be that an interaction between ATP1A1 and one or more RSV proteins occurs but is insufficiently stable to be detected by these methods. Using RSV deletion mutants, we did demonstrate that the RSV G protein is required to trigger ATP1A1 clustering. This implies that an initial virus attachment event involving G is needed to initiate ATP1A1 clustering. That initial attachment event might involve a direct interaction between RSV G and ATP1A1, although there is as yet no evidence of this, as already noted. It perhaps is more likely that RSV attachment is an earlier event involving other cellular structures, and that attachment induces ATP1A1 signaling through some as-yet unknown intermediate step. Clustering of signaling receptors in general can increase ligand binding and signal transduction [46] by reducing the effective dissociation rate through enhancing rebinding within the receptor cluster [47]. Therefore, clustering of ATP1A1 may be beneficial for RSV infection by enhancing the ATP1A1-mediated signaling that is required for viral uptake.

We hypothesized that RSV utilizes ATP1A1 signaling for uptake into the cell by endocytosis. This potentially could involve any of the various pathways including clathrin- or caveolin-mediated endocytosis or macropinocytosis. We showed that RSV infection indeed induces and requires ATP1A1 signaling, and confirmed that Src-kinase activity was required for efficient RSV infection. We also confirmed that EGFR is essential for efficient RSV infection, as previously shown [14], but is not sufficient alone and requires the upstream activation of ATP1A1 and c-Src for efficient RSV infection.

ATP1A1 signaling in response to ouabain results in clathrin-mediated endocytosis and the uptake and destruction of Na^+^,K^+^-ATPase [35, 36]. Clathrin-mediated endocytosis also has been controversially suggested to be involved in the uptake of RSV [15]. In preliminary experiments, inhibitors of clathrin-mediated endocytosis did not affect RSV infection. However, we found that RSV infection induced a high level of macropinocytosis, and that these macropinosomes contained a high content of RSV virions. Previous studies also have suggested a role for macropinocytosis in RSV uptake [14, 16]. Macropinocytosis also can be induced through components of the ATP1A1 signaling pathway. For example, it has been described that phosphorylation of EGFR Tyr845 by c-Src, in an EGF-independent manner, can lead to the induction of macropinocytosis [31, 32], Src-kinase activity plays an important role during macropinosome formation and trafficking [48], and can synergistically enhance macropinocytic induction [49].

Typical macropinosomes are formed as a result of extensive, nonspecific fluidic uptake at the plasma membrane (reviewed in [25, 33, 49]) that engulf fluid and solid cargo from outside of the cell into cytoplasmic vesicles. They are heterogeneous in size and are larger than other endocytic vesicles, with diameters of 0.5–5 μm. In RSV-infected cells, extensive macropinocytosis began very early in infection. Macropinosome formation under these conditions was confirmed to be dependent upon ATP1A1, and was significantly reduced if ATP1A1 expression was decreased or if the cells were treated with ouabain or PST2238. Depletion of cholesterol resulted in a decrease in the formation of macropinosomes, consistent with the involvement of signaling complexes in the caveolae. In addition, immunostaining revealed the co-localization of ATP1A1, RSV F protein (marker of viral envelope) and RSV N protein (marker of viral nucleocapsid), and dextran in macropinosomes. This supports a model in which RSV virions are taken up by the macropinosome, and membrane fusion and release of the nucleocapsid presumably taking place at a later step after the macropinocytic uptake. Although the data provide evidence on the role of ATP1A1 in macropinocytic uptake, they do not exclude the possibility of clathrin-mediated endocytosis as an additional mode of RSV entry.

It was somewhat surprising that both ouabain and PST2238 inhibited RSV infection in short-term treatments, since they have opposite effects on ATP1A1 signaling, namely that ouabain induces and PST2238 inhibits signaling. The mechanism by which PST2238 inhibits RSV infection seems straight-forward: specifically, blockade of RSV-induced ATP1A1 signaling. The mechanism by which ouabain inhibits RSV infection is less clear, since both ouabain and RSV individually induce ATP1A1 signaling. We hypothesize that, while the signaling cascades induced by ouabain versus RSV employ some of the same signaling intermediates, their outcomes are not exactly the same. Ouabain-induced signaling results in clathrin-mediated endocytosis, whereas RSV-induced signaling results in macropinocytosis. Also, we could readily detect phosphorylation of EGFR Tyr845 following infection with RSV, but not following treatment with ouabain, suggestive of a quantitative or qualitative difference in EGFR phosphorylation. Thus, while signaling through ATP1A1 by ouabain versus RSV may involve a number of steps in a common signal transduction pathway, we suggest that the ouabain-induced signaling cascade from ATP1A1 not only is different from that of RSV, but also competes with and thereby inhibits ATP1A1 signaling induced by RSV.

ATP1A1-mediated signaling cascades have been reported to take place in the cholesterol-rich microdomains called caveolae [27, 28], which are thought to serve as a region to integrate multiple signaling pathways by concentrating signaling proteins and creating temporal and spatial patterns of cell regulation [24]. Many proteins associated with signaling functions are present in the caveolae, including ATP1A1, EGFR and c-Src [17, 50, 51]. It has also been described that cholesterol is needed for the ouabain-induced ATP1A1-Src-EGFR signaling cascade, and that depletion of cholesterol reduced the recruitment of c-Src and therefore reduced ATP1A1 signaling [27]. Interestingly, it has been reported that the cholesterol-rich lipid rafts are required as a docking platform for RSV entry [9]. In the present study, depletion of cholesterol with MBCD and Mevinolin indeed reduced the efficiency of RSV infection, consistent with the signaling by ATP1A1, c-Src, and EGFR taking place in caveolae. We speculate that the inhibitory effects of depleting cholesterol could be due to disintegration of the caveolar ATP1A1-Src-EGFR signaling complexes.

A number of key observations made using the A549 cell line were reproduced using primary human small airway epithelial HSAEC cultures, including inhibition of RSV-GFP by siRNA as well as by PST2238 and Ouabain, clustering of ATP1A1 on RSV exposure, and phosphorylation of EGFR. We also used differentiated HAE-ALI cultures to confirm clustering of ATP1A1 on RSV infection, and to evaluate the distribution of ATP1A1 in a polarized epithelial cell. This showed a strong basolateral presence of ATP1A1 and much reduced speckled expression on the apical surface. However, upon RSV infection, ATP1A1 cluster formation was observed on the apical surface, associated with an increase in the apical amount of ATP1A1 that might be due to increased trafficking to the apical surface. This also showed that the small amounts of apical ATP1A1 present in the uninfected cells is likely sufficient for initiating the signaling cascade.

ATP1A1 also has been implicated as a pro-viral factor in the infection cycles of Ebola virus [52], coronavirus [53], hepatitis C virus [54], and mammarenaviruses [55], but the nature and mechanism of its involvement for those viruses remains largely unknown. In the present study, we showed that VSV infection was not inhibited by ATP1A1 knockdown, indicating that the effect is specific to particular viruses and does not involve a general inhibitory cellular effect. Ouabain has been described to have anti-viral properties for several viruses, namely herpes simplex virus [56, 57], chikungunya virus [58], human immunodeficiency virus [59], adenovirus [60] and porcine reproductive and respiratory syndrome virus [61], but the mechanism of inhibition was not conclusively identified. It may be that ouabain inhibits these other viruses in the same way that we propose for RSV, namely that it competes with the virus for signaling through ATP1A1. To our knowledge, PST2238 has not previously been investigated for anti-viral activity against any virus, including RSV. We speculate that PST2238 might also inhibit the replication of other viruses that are ouabain-sensitive by directly blocking ATP1A1 signaling. PST2238 is being evaluated as an anti-hypertensive drug in phase II clinical trials for ouabain- and adducin-induced hypertension patients [38]. PST2238 does not have any known adverse effects, does not lower the blood pressure of healthy humans [62], and therefore might be well-tolerated as an anti-viral drug.

The prevailing paradigm of virus spread involves packaging, release, and infection of the neighboring cells. However, an emerging model suggests direct cell-to-cell spread of partially assembled virions, nucleocapsids, and inclusion bodies via intercellular channels [63]. Given the very different nature of this exit/entry, likely requiring a different set of proteins and mechanisms, we speculate that ATP1A1 may not have a role in this and would be primarily engaged in mediating uptake of the extracellular packaged virions. The proposed direct intercellular spread presumably occurs only between adjoining cells, while ATP1A1 would be important for spread/uptake into non-contiguous cells. Note that most studies have described the direct intercellular transmission using cell lines (reviewed in [63]). Specifically, among respiratory viruses, intercellular transmission in primary HAE cells has been shown only for measles virus [64] and this manner of spread for other respiratory viruses including RSV remains to be determined in a primary differentiated HAE-ALI system.

**Fig 13:**
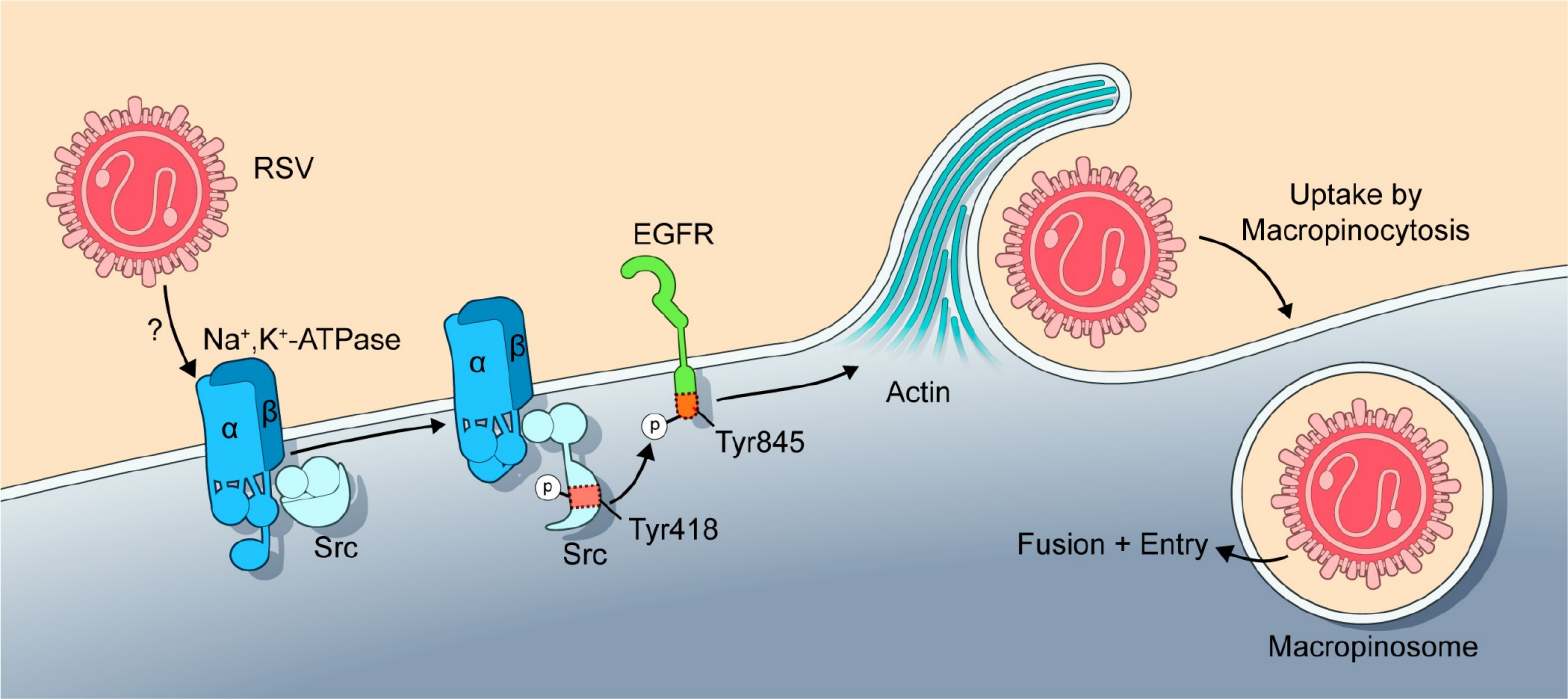
Proposed model of ATP1A1-dependent macropinocytic entry of RSV. On exposure to respiratory epithelial cells, RSV triggers the activation and clustering of ATP1A1 in the plasma membrane through an unknown mechanism. ATP1A1 then signals via phosphorylated Src-kinase and transactivates EGFR by its phosphorylation at Tyr845. Upon activation, EGFR signaling causes cytoskeletal rearrangement resulting in plasma membrane ruffling and formation of membrane extensions (reported previously [31, 33, 34]) that engulf fluid and RSV into macropinosomes. Note that RSV is taken up into the macropinosome in its enveloped state suggesting that it does not fuse at the cell surface; fusion and release of the nucleocapsid likely occurs within the internalized macropinosome.

In summary, we propose a model for RSV entry into human airway epithelial cells that is illustrated in Fig 13. RSV infection activates ATP1A1 signaling by an unknown mechanism that involves the RSV G protein and does not rely on viral transcription and genome or virus replication. Activation of ATP1A1 leads to transactivation of EGFR that requires c-Src kinase activity. Signaling events downstream of EGFR cause actin rearrangement and ruffling at the plasma membrane, where membrane extensions engulf fluid and RSV into large vesicles—the macropinosomes. RSV is taken up in its enveloped form into the macropinosome, presumably followed by fusion and entry into the host cell. We also provided evidence suggesting that the ATP1A1-Src-EGFR signaling occurs predominantly in the cholesterol-rich domains of the caveolae which are thus important for efficient infection. This study identified ATP1A1 signaling as a new target for the development of anti-RSV drugs. PST2238 in particular is a drug that already has been shown to be well-tolerated in humans, specifically acts on ATP1A1, decreases the efficiency of infection by reducing the RSV-ATP1A1 signaling needed for entry, and could be further developed as an antiviral drug for RSV.

## Materials and methods

### Cells and viruses

A549 cells (ATCC CCL-185) were maintained in F12-K media (ATCC, Manassas, VA) supplemented with 10% fetal bovine serum (FBS, Thermo Scientific, Atlanta, GA) and 1x L-Glutamine (Life Technologies, Grand Island, NY), Vero cells (ATCC CCL-81) were maintained in Opti-MEM I medium with GlutaMax-I (Life Technologies) supplemented with 5% FBS. Normal primary human small airway epithelial cells (HSAEC) (ATCC PCS-301-010) were derived from a 16 year old male Hispanic/Latino donor (Lot: 64079184) and were maintained in airway cell basal medium (ATCC PCS-300-030), supplemented with bronchial epithelial cell growth kit (ATCC PCS-300-040), and detached for passage using trypsin and trypsin-neutralizing solution formulated specifically for primary cells (ATCC PCS-999-003 and ATCC PCS-999-004. The primary cells were passaged a maximum of two times. HAE-ALI cultures (EpiAirway, AIR-100) were obtained from MatTek Corporation (Ashland, MA) and were cultured at the air-liquid interface as described in the manufacturer’s protocol with the provided maintenance medium, with daily medium changes.

The recombinant viruses RSV-GFP [65], VSV-GFP [66], wt RSV A2 (Genbank accession # KT992094), rgRSV-dSH and rgRSV-dSH/dG [7] have been previously described. For all experiments, virus stocks were purified on a discontinuous (60% and 30% w/v) sucrose gradient as described previously [65]. Cell cultures were maintained at 37ºC and 5% CO_2_, and any cells used in experiments were incubated under these conditions.

### Inhibitors and chemical compounds

The chemical compounds and inhibitors ouabain (PubChem CID: 439501), PST2238 (rostafuroxin, PubChem CID: 153976), Src-Inhibitor-I (PubChem CID: 1474853), PP2 (PubChem CID: 4878), methyl-beta-cyclodextrin (MBCD, PubChem CID: 51051622) and Mevinolin (Lovastatin, PubChem CID: 53232) were obtained from Sigma-Aldrich, St. Louis, MO. 50 μM ouabain stock solution was prepared in sterile ultrapure water. 10 mM stock solutions of PST2238, Src-Inhibitor-I and PP2 were prepared in DMSO. 76.3 mM MBCD stock solution was prepared in F12 media. 1 mg/ml Mevinolin stock solution was prepared in 200 proof ethanol. Working stock solutions, at concentrations as indicated, were prepared in the appropriate cell culture media. Non-cytotoxic concentrations for all chemical compounds were determined by serial dilution on A549 cells (see S3 Fig) and the cytotoxicity was quantified by the ATP-based viability assay, as described below. The final DMSO concentration was below 0.2% and was considered not to have any effect on the cells as determined by DMSO control treated cells (S3 Fig).

### siRNA screen

The high-throughput screen was performed and the data were analyzed as described previously [67]. In brief, the siRNAs were from the Ambion Silencer Select Human Genome siRNA library version 4 (Fischer Scientific), targeting ~21,500 human genes with 3 siRNAs per gene, evaluated individually. High throughput screening was performed in 384-well plates (total of 186 plates) using a Bravo VPrep liquid handler within a BioCel automation platform (Agilent). Single siRNAs (0.8 pmol) were added to wells followed by 20 μl of serum-free medium containing 0.12 μl Lipofectamine RNAiMax. The mixtures were incubated for 45 min at room temperature, followed by the addition of A549 cells that we had engineered to constitutively express DsRed as a viability marker. The final transfection mixtures contained 1000 A549 cells and 20 nM siRNA in F12 medium and 10% FBS. The cells were incubated for 48 h, RSV-GFP was added at a multiplicity of infection (MOI) of 1 PFU/cell, and cells were incubated for an additional 48 h. The cells were fixed with paraformaldehyde and stained with Hoechst 33342. Images were acquired on an ImageXpress Micro XL (Molecular Devices) automated microscope and analyzed with associated MetaXpress software using the “Multi Wavelength Cell Scoring” analysis module. Ambion Silencer Select Negative Control #2 was present in all screening plates (16 wells) and the median negative control value on each plate was used for normalization. An siRNA targeting GFP was used as a positive control (16 well per plate) and yielded near-complete elimination of cells scoring positive for GFP. The median absolute deviation (MAD) based z-score was calculated for each siRNA [68]. Scores were adjusted for seed-based off-target effects to help deprioritize likely false positives [69]. The median seed-adjusted Z-scores are provided in the S1 Table.

### siRNA transfection to knock down the host proteins ATP1A1 and EGFR

A549 and HSAEC cells were transfected with siRNA by reverse transfection with siLentFect transfection reagent (Bio-Rad, Hercules, CA) in 12-well plates. For the ATP1A1 and EGFR knock down the following siRNAs (obtained from Qiagen, Germantown, MD) were used: Hs_ATP1A1_5 (named siRNA1, CCC GGA AAG ACT GAA AGA ATA), Hs_ATP1A1_6 (named siRNA2, CTT GAT GAA CTT CAT CGT AAA), Hs_ATP1A1_7 (named siRNA3, ATC CAT GAA GCT GAT ACG ACA), Hs_EGFR_3 (named EGFR siRNA, CAG AGG AAA TAT GTA CTA CGA). These were different from those used in the high throughput screen. The two negative control siRNAs were Neg. siRNA 1 (AllStars Neg. Conrol siRNA [Qiagen 1027281, sequence proprietary]) and Neg. siRNA 2 (Negative Control siRNA (Qiagen 1027310, AAT TCT CCG AAC GTG TCA CGT). Transfection methods were optimized with the cell death positive control siRNA (AllStars Hs Cell Death siRNA control [Qiagen 1027299]).

### Cell viability assay

Cell viability was measured by an ATP-based assay CellTiter-Glo (Promega, Madison, WI) performed as described by the manufacturer. Cells in white 96-well plates were lysed and the ATP concentration was determined by luciferase activation. Luciferase light emission was analyzed using a Synergy 2 ELISA reader (BioTek, Winooski, VT). Reduction in ATP relative to control cells was indicative of reduced viability.

### Western blot analysis for the quantification of ATP1A1

A549 or HSAEC cells in 12-well plates were lysed with 75 μl 1x LDS sample buffer (Life Technologies). 22.5 μL aliquots of lysate were reduced, denatured, and electrophoresed on 4–12% Bis-Tris SDS gels (Life Technologies). Proteins were transferred onto PVDF membranes using the iBlot2 transfer system (Life Technologies) and analyzed by Western blotting. ATP1A1 was detected with an anti-ATP1A1 rabbit MAb (Abcam, Cambridge, MA; ab76020) and the corresponding infrared dye-conjugated goat anti-rabbit immunoglobulin 680RD (Li-Cor, Lincoln, NE). Tubulin was used as a loading control and was detected with an anti-tubulin mouse MAb and an infrared dye-conjugated goat anti-mouse immunoglobulin 800CW (Li-Cor). Western blot images were acquired on the Odyssey infrared scanner (Li-Cor) and analyzed with Image Studio Software (Version 5.2.5, Li-Cor). ATP1A1 band intensity values were normalized to tubulin and reported relative to Neg. siRNA1 transfected cells.

### Quantitative RT-PCR

Cells were harvested and total RNA was isolated with RNeasy Mini Kit (Qiagen) as described by the manufacturer’s protocol, including on-column DNase digestion. 1 μg total RNA was used for reverse transcription with oligo(dT) _12–18_ primers and the SuperScript TM First-Strand Synthesis System for RT-PCR (Life Technologies). The synthesized cDNA was pre-diluted 1:10 and used for the TaqMan gene expression analysis of ATP1A1 (Hs00167556_m1) and 18S rRNA (Hs99999901_s1) as a normalization control. The TaqMan assay reactions were analyzed on the 7900HT Fast Real-Time PCR system (Applied Biosystems, Foster City, CA). The threshold cycle (Ct) for each reaction was determined by the SDS RQ Manager program (Applied Biosystems). The relative changes in ATP1A1 transcript level were calculated by the 2^-ΔΔCt^ method [70] and reported relative to cells transfected with Neg. siRNA 1.

### Quantification of RSV gene expression by viral expressed GFP

RSV gene expression was measured using RSV-GFP, a recombinantly derived virus that expresses enhanced green fluorescent protein (eGFP) from an additional gene inserted between the P and M genes. SiRNA transfected or pre-treated A549 cells in 12-well plates were inoculated with an MOI of 1 PFU/cell. Inoculum was adsorbed for 2 h on a rocking platform at 37°C and then washed off and replaced with fresh media. The infected cells were incubated for 17 h, and GFP expression was quantified either by ELISA reader or flow cytometry. For the ELISA reader quantification, the GFP intensity of the infected monolayer was quantified by an area scan (average GFP intensity of 29 individual measurements per well) on a Synergy 2 ELISA reader (BioTek). The intensity values were adjusted by subtracting the average intensity of mock-infected cells as background and reported relative to Neg. siRNA 1 transfected or mock-treated cells that had been infected with RSV-GFP. For the flow cytometry-based GFP quantification, the cells were detached with 1 mM EDTA, stained with LIVE/DEAD fixable dead cell staining kit (Life Technologies), and fixed with 4% paraformaldehyde (PFA, Electron Microscopy Science, PA). The GFP intensities of single, live cells were analyzed on a Canto II flow cytometer (BD Biosciences, Franklin Lakes, NJ). The median fluorescence intensity (MFI) of GFP-positive cells was determined and reported as change relative to Neg. siRNA 1 transfected or mock-treated cells that had been infected with RSV-GFP.

### Virus titration

Infected cell monolayers were scraped into the media supernatant, vortexed for 30 s, clarified by centrifugation at 478 x g, snap frozen on dry-ice, and stored at -80°C. Samples were 10-fold serially diluted and Vero cells were inoculated with each dilution in duplicate and incubated for 2 h on a rocking platform at 37°C. Cells were overlaid with OptiMEM I (Life Technologies) containing 0.8% methylcellulose (Sigma-Aldrich), 1x L-Glutamine, 2% FBS and 50 μg/ml Gentamicin and incubated for 6 days at 37°C. For RSV that expressed GFP, the plaques were visualized directly by GFP expression with a Typhoon imaging system (GE Healthcare, Chicago, IL). Wt RSV plaques were fixed with ice-cold 80% methanol and immunostained with a mix of three RSV F-specific mouse MAbs [71] followed by a 680RD infrared dye-conjugated goat anti-mouse secondary immunoglobulin (Li-Cor). The plaques were imaged on the Odyssey infrared scanner (Li-Cor) and were counted using the ImageJ software (Version 1.46r; NIH, Bethesda, MD).

### Immunofluorescence microscopy

A549 cells were seeded on glass cover slips in 24-well plates and used when still sub-confluent. For the immunofluorescence microscopy based assays, cells were fixed with 4% paraformaldehyde for 16 h at 4°C, permeabilized with 0.1% TritonX-100 (Sigma Aldrich) for 15 min and blocked with PBS containing 5% bovine serum albumin (BSA) (Sigma Aldrich) for 1 h at room temperature. All antibody dilutions were prepared in PBS, containing 5% BSA and 0.1% TritonX-100. The primary antibody incubation was performed in a humidified chamber for 2 h with the following antibodies: rabbit anti-ATP1A1 (Abcam; ab76020, 1:100), rat anti-EGFR (Abcam; ab231, 1:100), mouse anti-RSV-N (Abcam; ab94806, 1:1,500) and anti-RSV-F (mouse MAb #1129 [72], 1:200). After washing with PBS, the secondary antibody staining was performed with the respective Alexa Fluor (AF) conjugated secondary antibodies: donkey anti-rabbit AF488, goat anti-rabbit AF700, donkey anti-mouse AF647, goat anti-rat AF647. Simultaneous staining of RSV-F and RSV-N was performed with conjugated primary antibodies: specifically, anti-RSV-F mouse MAb #1129 [72] that we conjugated with AF488 [Antibody Labeling Kit (Thermo Fisher Scientific, Waltham, MA)]; and a commercially-available anti-RSV N mouse MAb conjugated with allophycocyanin (APC) (NB100-64752APC, Novus Biologicals, Littleton, CO). Nuclei were counterstained with 300 nM DAPI (Life Technologies) in PBS for 5 min and mounted on glass-slides with ProLong Diamond Antifade mountant (Life Technologies). Immunostainings of HAE-ALI cells were performed as described above for A549 cells, except the incubation times of the primary and secondary antibodies were extended to 16 h at 4°C. In addition, the cultures were stained for F-Actin with the SiR-actin kit (CY-SC001; Cytoskeleton; Inc, Denver, CO). Images were acquired on a Leica TCS-SP8 confocal microscope (Leica Microsystems, Mannheim, Germany) using a 63x oil immersion objective (NA 1.4), a zoom between 1.0 to 4.0x, laser emission at 488nm for AF488, 561 nm for AF568 and 633 nm for AF647. DAPI was excited using a 450 nm diode laser. Detector slits were configured to minimize any crosstalk between the channels and, if necessary, the channels were collected sequentially and merged afterwards. Images were processed using Leica Application Suite X (LAS-X) software, Imaris (Version 9.0.0, Bitplane AG, Zurich, Switzerland) and ImageJ. Images of the HAE-ALI cells were deconvolved with Huygens Essential deconvolution software (Scientific Volume Imaging B.V, Hilversum, The Netherlands).

### Quantification of EGFR phosphorylation

An EGFR phosphorylation array (Ray Biotech, Norcross, GA) was used to analyze phosphorylation of the EGFR receptor family. The array consisted of nitrocellulose membrane that was spotted in duplicate with phospho-specific antibodies against 17 different phosphorylation sites of the human EGFR family, plus a positive control antibody that binds EGFR irrespective of phosphorylation. Cells were treated as indicated, either transfected with siRNA 48 h prior or pre-treated with the indicated chemical compound for 16 h. Coincident with transfection or treatment, the cells were serum-starved for 16 h and then infected with wt RSV (MOI = 5 PFU/cell) for 5 h at 37°C. Cells treated with Ouabain or PST2238 for 16 h were incubated in F12 medium containing EGF (Sigma-Aldrich, 100 ng/ml) for 45 min as controls for PST2238 and Ouabain specificity. Cells were washed twice with cold PBS and lysed in the provided lysis buffer, containing protease and phosphatase inhibitor cocktails. The protein concentration of the lysate was quantified by bicinchoninic acid (BCA) assay (Thermo Fisher Scientific) and lysate containing 150 μg total protein was used for each array. The array was processed as described by the manufacturer, in brief: the array was incubated with the diluted lysate for 16 h at 4°C on a rocking platform, washed, and incubated with a biotinylated pan EGFR antibody followed by horseradish peroxidase-conjugated streptavidin, and luminescence was detected with X-ray film. The films were scanned and spot intensity was quantified by ImageQuant TL (Array Version 8.1., GE Healthcare). Three independent experiments with two technical replicates each were normalized to the internal array controls and pan EGFR. Values are reported relative to the average signal of Neg. siRNA 1 (for siRNA-transfected) or to mock-treated (for inhibitor-treated), RSV-infected samples.

### Dextran uptake assay for macropinocytosis

Following a published procedure [73], A549 cells on cover slips were transfected with siRNA for 48 h or were treated with indicated chemical compound. Coincident with transfection or treatment, the cells were serum-starved for 16 h. The cells were infected with wt RSV (MOI = 5 PFU/cell) in media containing AF568-conjugated dextran (Dextran-AF568; 10,000 MW; Life Technologies), incubated for 5 h at 37°C, and washed and fixed for 16 h with 4% PFA at 4°C. For co-localization experiments, the cells also were stained for ATP1A1, RSV F, and RSV N using specific antibodies as described above. Nuclei were counterstained with DAPI (300 nM in PBS for 5 min) and mounted on glass slides with ProLong Diamond Antifade mountant. For each treatment, at least ten random images were acquired, using the mark and find function of the Leica LAS-X image acquisition software, as Z-stacks on a Leica TCS-SP8 confocal microscope (Leica) with a 63x Objective (NA 1.4) and a zoom of 1.0x. The images were analyzed by batch process with the software Imaris (Version 9.0.0, Bitplane AG). The DAPI-stained nuclei were detected as spots to count the number of cells per field. The uptake of dextran was quantified by using the surface function of Imaris to recognize distinct dextran-positive vesicles. The total intensity of dextran-AF568 within the created surfaces, which had a volume larger than 1.0 μm^3^, of one field was normalized to the number of nuclei per field. The values were reported relative to Neg. siRNA 1-transfected or mock treated cells that had been infected with RSV. For each experiment a total of at least 600 cells per condition were analyzed.

### Ethics statement

All animal studies were approved by the NIH Institutional Animal Care and Use Committee (IACUC) under the animal study protocol number LID 34E. The National Research Council’s Guide for the care and use of laboratory animals and the Public Health Service Policy on humane care and use of laboratory animals served as the guidelines for the care and use of mice in this study.

The primary HSAEC and HAE-ALI cells were obtained from the American Type Culture Collection (ATCC) and MatTek Corporation, respectively. The cells were isolated under informed consent and conform to HIPAA standards to protect the privacy of the donor’s personal health information. The research with these human cells is not considered human subjects research because the cells are publicly available and therefore was exempt from 45 CFR Part 46 and no Institutional Review Board (IRB) approval was required.

## Supporting information

S1 Table Summary of the primary siRNA screen. The expression knock-down siRNA screen was performed by transfecting A549 cells, engineered to express DsRed as a viability marker, with the Ambion Silencer Select Genome-wide siRNA library V4 targeting 21,566 genes, with three independent siRNAs per gene evaluated individually. Forty-eight h post-transfection the cells were infected with 1 PFU/cell of RSV-GFP. The effect of knock-down on GFP expression was measured 48 h post-infection, and the data analyzed as previously described [67]. The median seed corrected Z-scores for all the tested targets is shown.
(XLSX)

S1 Fig Time course of ATP1A1 knock down by siRNA transfection. (**A-C**) See legend and description of Fig 1. **(D) Cell viability.** An ATP-based cell viability assay [CellTiter-Glo (Promega)] was performed 72 h p.t. to evaluate the viability of the transfected cells. Cells were lysed, the ATP concentration was determined by ATP-dependent luciferase activity, which was detected with an ELISA reader, and the viability was reported relative to mock-transfected cells assigned the value of 1.0.
(TIF)

S2 Fig ATP1A1 clustering induced by UV-inactivated RSV. A549 cells were inoculated (MOI = 5 PFU/cell) as described for Fig 3 and incubated for 5 h. The UV wt RSV inoculum was UV-inactivated by 0.5 J/cm^2^ UV radiation using a Stratalinker UV Crosslinker 1800 (Agilent). Total inactivation of the inoculum was confirmed by plaque titration on Vero cells. Cells were subjected to immunofluorescence staining for ATP1A1 (AF488, green), RSV-N (AF568, red), and counterstained the nuclei with DAPI (blue). Scale bars 10 μm.
(TIF)

S3 Fig Anti-viral efficacy (IC_50_) and cytotoxicity of ouabain and PST2238 on A549 cells and primary human small airway epithelial cells (HSAEC). **(A, B) Antiviral efficacy**. A549 cells (solid line) and HSAEC (dotted line) were treated with the indicated concentrations of ouabain (A) and PST2238 (B) for 5 h, infected with RSV-GFP (MOI = 1 PFU/cell), and incubated for 24 h in the continued presence of the respective drug. Each combination of cell type and drug concentration was done in triplicate. GFP intensity as an indicator of viral infection was measured by scanning each well completely with an ELISA reader and reported relative to mock-treated infected cells set at 1.0, with error bars indicating the standard deviation.
**(C, D) Cytotoxicity**. A549 cells (solid line) and HSAEC (dotted line) were incubated with the indicated concentrations of ouabain (C) and PST2238 (D) for 24 h in triplicates for each combination of cell type and drug concentration. Viability was assessed by the ATP-based viability assay CellTiterGlo (Promega), and the results were expressed relative to mock-treated cells assigned the value of 1.0, with error bars indicating the standard deviation. The horizontal dotted line indicates 80% viability.
(TIF)

S4 Fig Cytotoxicity of chemical compounds on A549 cells. A549 cells were treated for 24 h with each compound at the highest concentrations used in this study. Cell viability was determined in triplicates for each compound by the ATP-based viability assay CellTiterGlo (Promega), and the results were expressed relative to mock-treated cells assigned the value of 1.0, with error bars indicating the standard deviation.
(TIF)

S5 Fig siRNA knock down of EGFR. A549 cells were transfected with an EGFR-specific siRNA and at 48 h p.t. the cells were lysed in 1x LDS buffer. The lysates were subjected to Western blot analysis with an EGFR-specific mouse MAb (ab181822; Abcam) and a corresponding IRDye 680RD-conjugated goat anti-mouse secondary antibody. Alpha-tubulin was used as loading control and was detected by an anti-alpha-tubulin mouse MAb and the same secondary antibody as EGFR.
(TIF)

S6 Fig RSV-induced phosphorylation of EGFR and EGFR family proteins (ErbB2, ErbB3, and ErbB4). **(A)** X-ray films of two complete EGFR phosphorylation-specific antibody arrays, probed with uninfected (left) or RSV-infected (right) A549 cell lysates as indicated. This is from the experiment described in Fig 8, which shows selected X-ray film spots from the complete set of arrays. **(B)** Layout of the EGFR phospho-specific antibodies and the control spots on the array (RayBiotech). Each antibody is present in duplicate on each membrane, as shown.
(TIF)

S7 Fig RSV-induced macropinocytosis very early during infection. Earlier timepoints (30 min and 1 h p.i.) of the experiment shown in Fig 9B
(TIF)

S8 Fig Effects on RSV-GFP expression and cell viability of chlorpromazine as an inhibitor of clathrin-mediated endocytosis. A549 cells were pre-treated for 5 h with serially-diluted concentrations of chlorpromazine and inoculated with RSV-GFP (MOI = 1 PFU/cell) while the inhibitor was continuously present. GFP was quantified by an ELISA reader 17 h p.i. (solid line) and cell viability was evaluated for each concentration by the ATP-based viability assay CellTiter-Glo (dotted line). GFP-intensity and luciferase activity was reported relative to mock-treated cells assigned the value of 1.0, with error bars indicating the SD. The dashed horizontal line reflects 80% viability.
(TIF)

S1 Movie Cross-section of A549 cell showing ATP1A1 clustering on the plasma membrane during RSV infection 5 h p.i. (shown in Fig 3A). The image was acquired as described for Fig 3. 0.3 μm Z-sections of the entire cell volume were acquired, combined to a three-dimensional image in Imaris (Version 9.0.0, Bitplan AG), and the video displays the ZY-view. Scale bar 10 μm.
(MOV)
