# Supplementary figures and images for: The alpha-1 subunit of the Na+,K+-ATPase (ATP1A1) is required for macropinocytic entry of respiratory syncytial virus (RSV) in human respiratory epithelial cells
Source: PLoS Pathog. 2019 Aug 5;15(8):e1007963. doi: 10.1371/journal.ppat.1007963 (PMC6695199; doi:10.1371/journal.ppat.1007963)

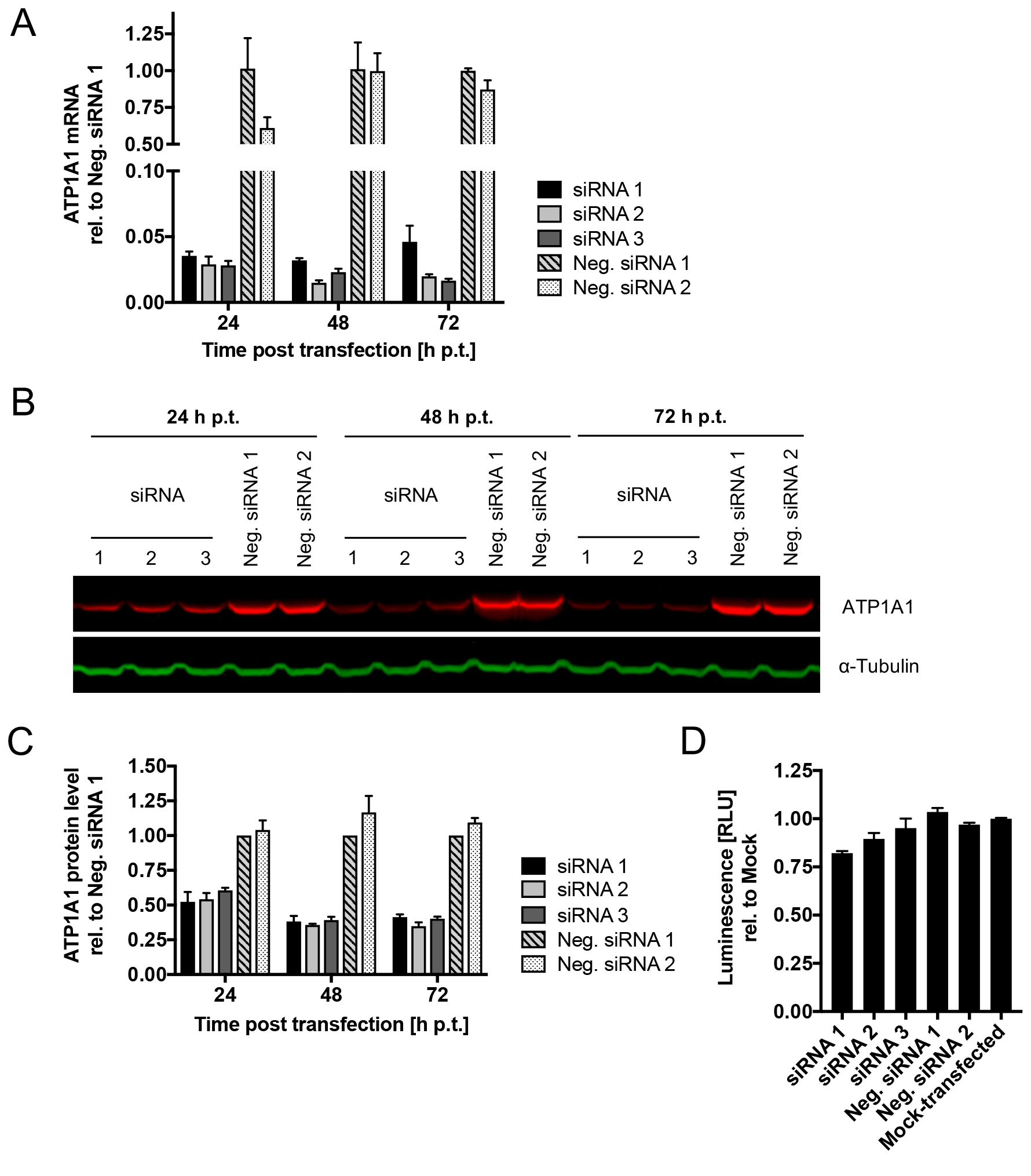

Supplement: S1 Fig — (A-C) See legend and description of Fig 1. (D) Cell viability. An ATP-based cell viability assay [CellTiter-Glo (Promega)] was performed 72 h p.t. to evaluate the viability of the transfected cells. Cells were lysed, the ATP concentration was determined by ATP-dependent luciferase activity, which was detected with an ELISA reader, and the viability was reported relative to mock-transfected cells assigned the value of 1.0. (TIF) [file ppat.1007963.s002.tif]

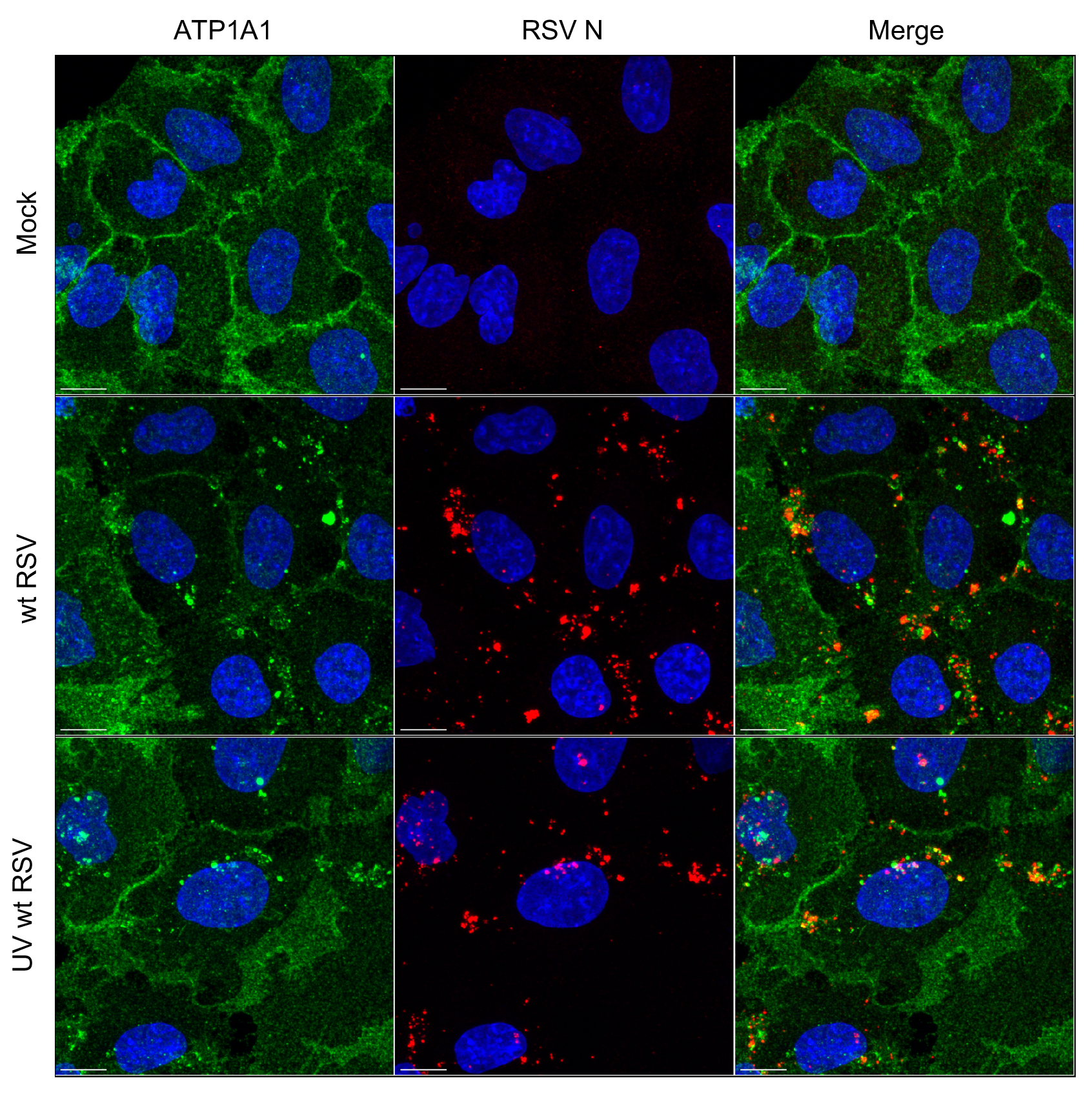

Supplement: S2 Fig — A549 cells were inoculated (MOI = 5 PFU/cell) as described for Fig 3 and incubated for 5 h. The UV wt RSV inoculum was UV-inactivated by 0.5 J/cm2 UV radiation using a Stratalinker UV Crosslinker 1800 (Agilent). Total inactivation of the inoculum was confirmed by plaque titration on Vero cells. Cells were subjected to immunofluorescence staining for ATP1A1 (AF488, green), RSV-N (AF568, red), and counterstained the nuclei with DAPI (blue). Scale bars 10 μm. (TIF) [file ppat.1007963.s003.tif]

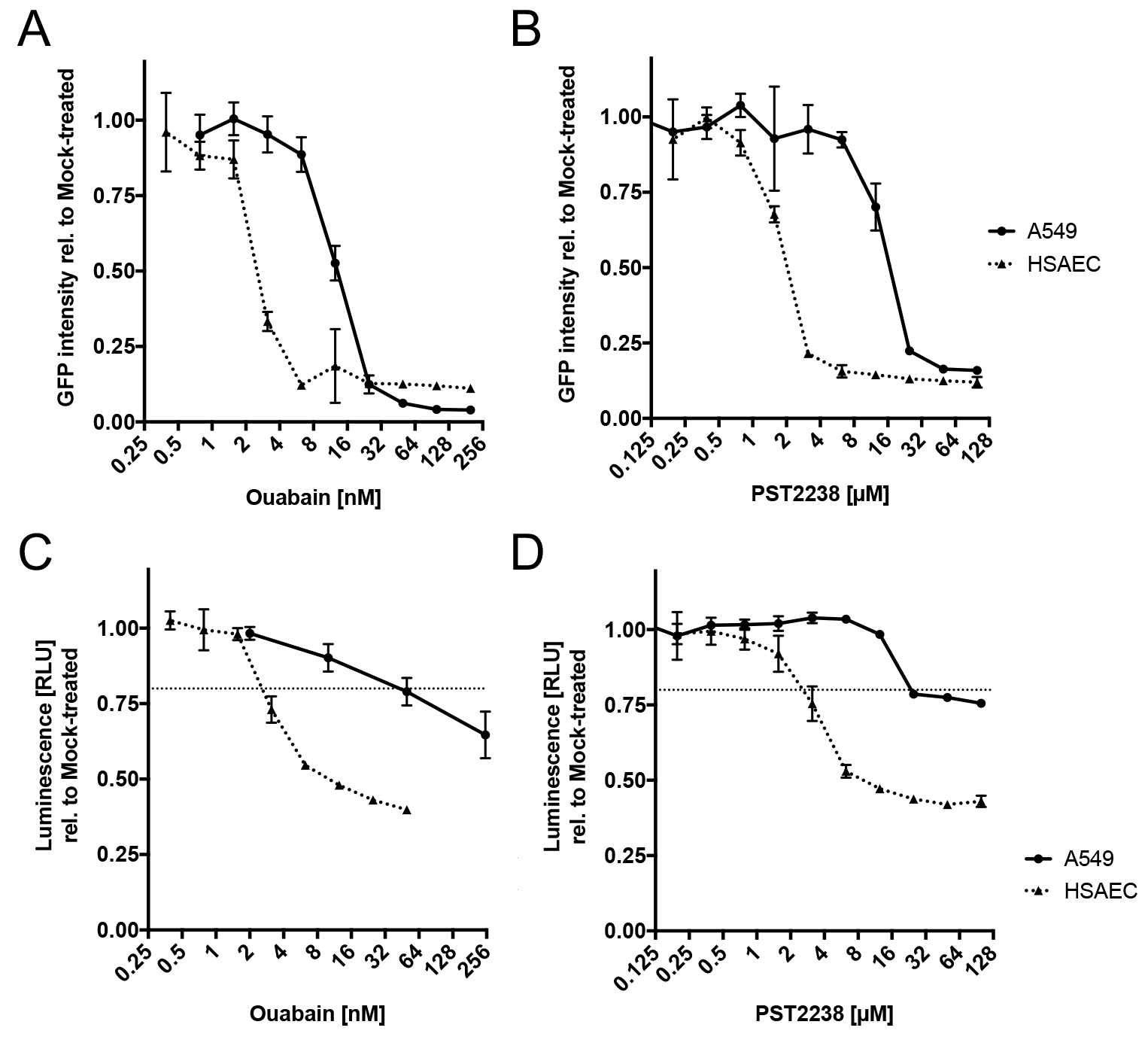

Supplement: S3 Fig — (A, B) Antiviral efficacy. A549 cells (solid line) and HSAEC (dotted line) were treated with the indicated concentrations of ouabain (A) and PST2238 (B) for 5 h, infected with RSV-GFP (MOI = 1 PFU/cell), and incubated for 24 h in the continued presence of the respective drug. Each combination of cell type and drug concentration was done in triplicate. GFP intensity as an indicator of viral infection was measured by scanning each well completely with an ELISA reader and reported relative to mock-treated infected cells set at 1.0, with error bars indicating the standard deviation. (C, D) Cytotoxicity. A549 cells (solid line) and HSAEC (dotted line) were incubated with the indicated concentrations of ouabain (C) and PST2238 (D) for 24 h in triplicates for each combination of cell type and drug concentration. Viability was assessed by the ATP-based viability assay CellTiterGlo (Promega), and the results were expressed relative to mock-treated cells assigned the value of 1.0, with error bars indicating the standard deviation. The horizontal dotted line indicates 80% viability. (TIF) [file ppat.1007963.s004.tif]

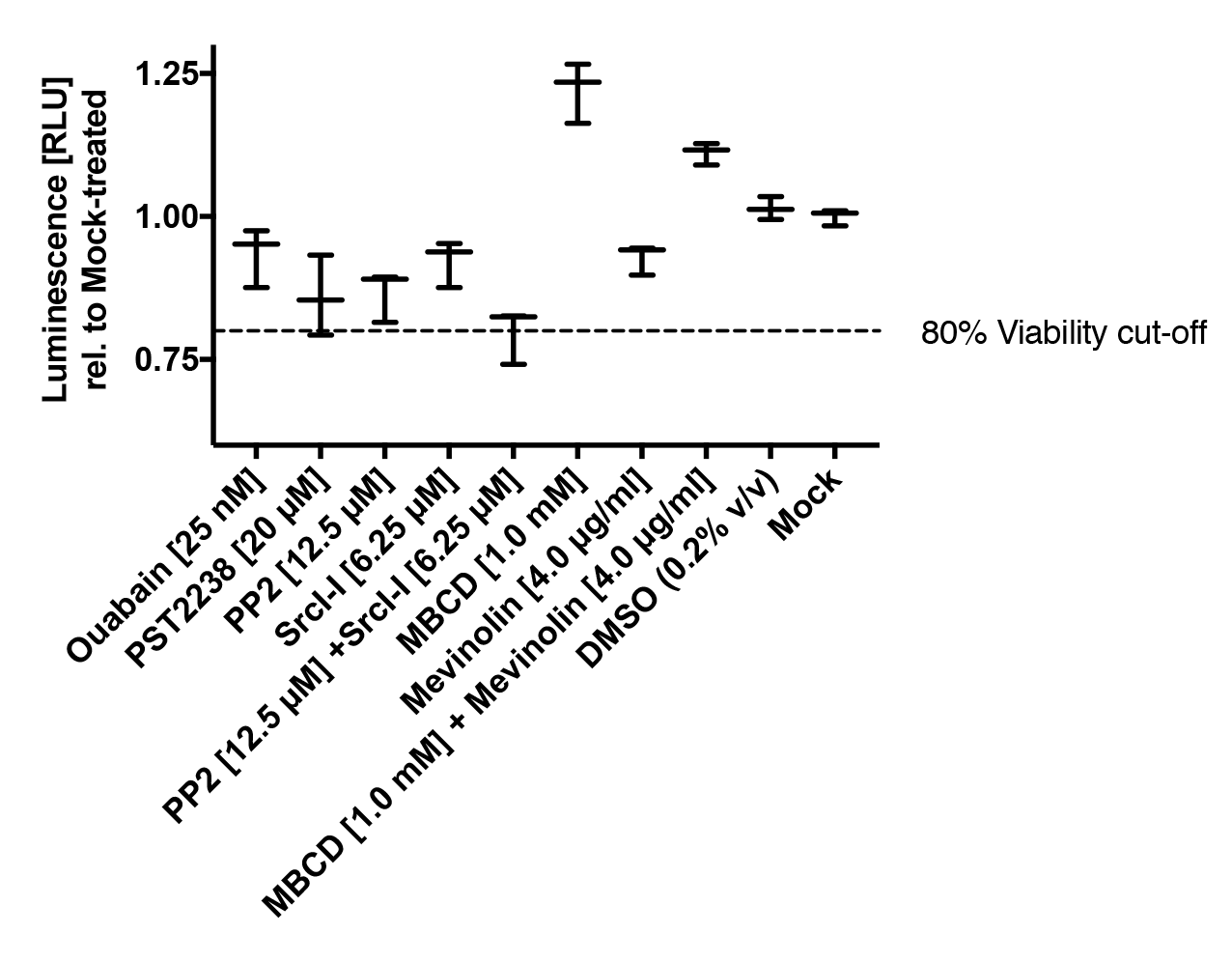

Supplement: S4 Fig — A549 cells were treated for 24 h with each compound at the highest concentrations used in this study. Cell viability was determined in triplicates for each compound by the ATP-based viability assay CellTiterGlo (Promega), and the results were expressed relative to mock-treated cells assigned the value of 1.0, with error bars indicating the standard deviation. (TIF) [file ppat.1007963.s005.tif]

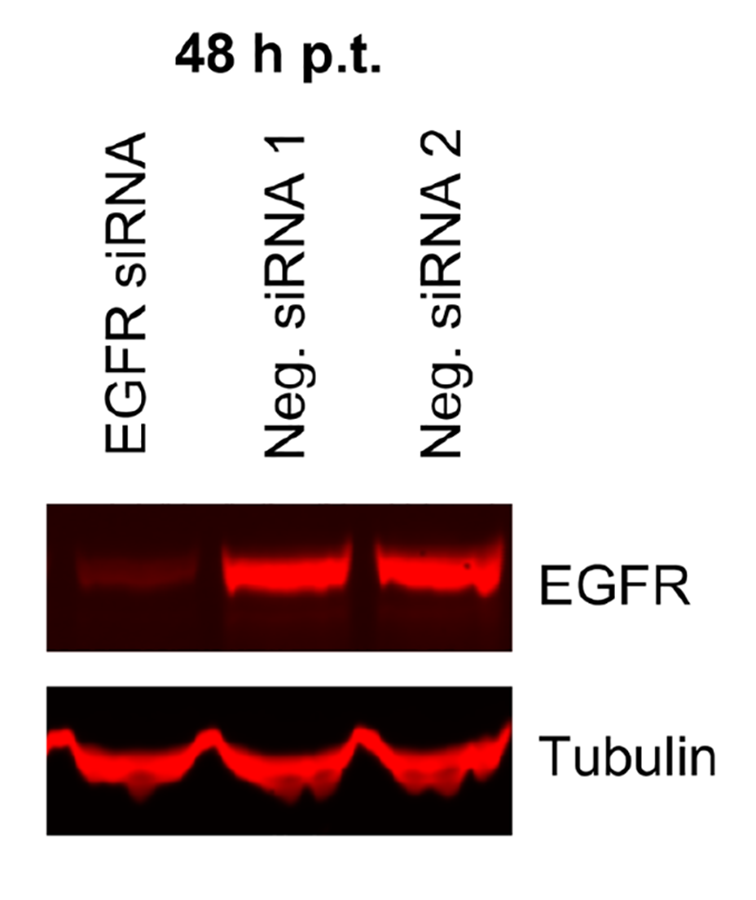

Supplement: S5 Fig — A549 cells were transfected with an EGFR-specific siRNA and at 48 h p.t. the cells were lysed in 1x LDS buffer. The lysates were subjected to Western blot analysis with an EGFR-specific mouse MAb (ab181822; Abcam) and a corresponding IRDye 680RD-conjugated goat anti-mouse secondary antibody. Alpha-tubulin was used as loading control and was detected by an anti-alpha-tubulin mouse MAb and the same secondary antibody as EGFR. (TIF) [file ppat.1007963.s006.tif]

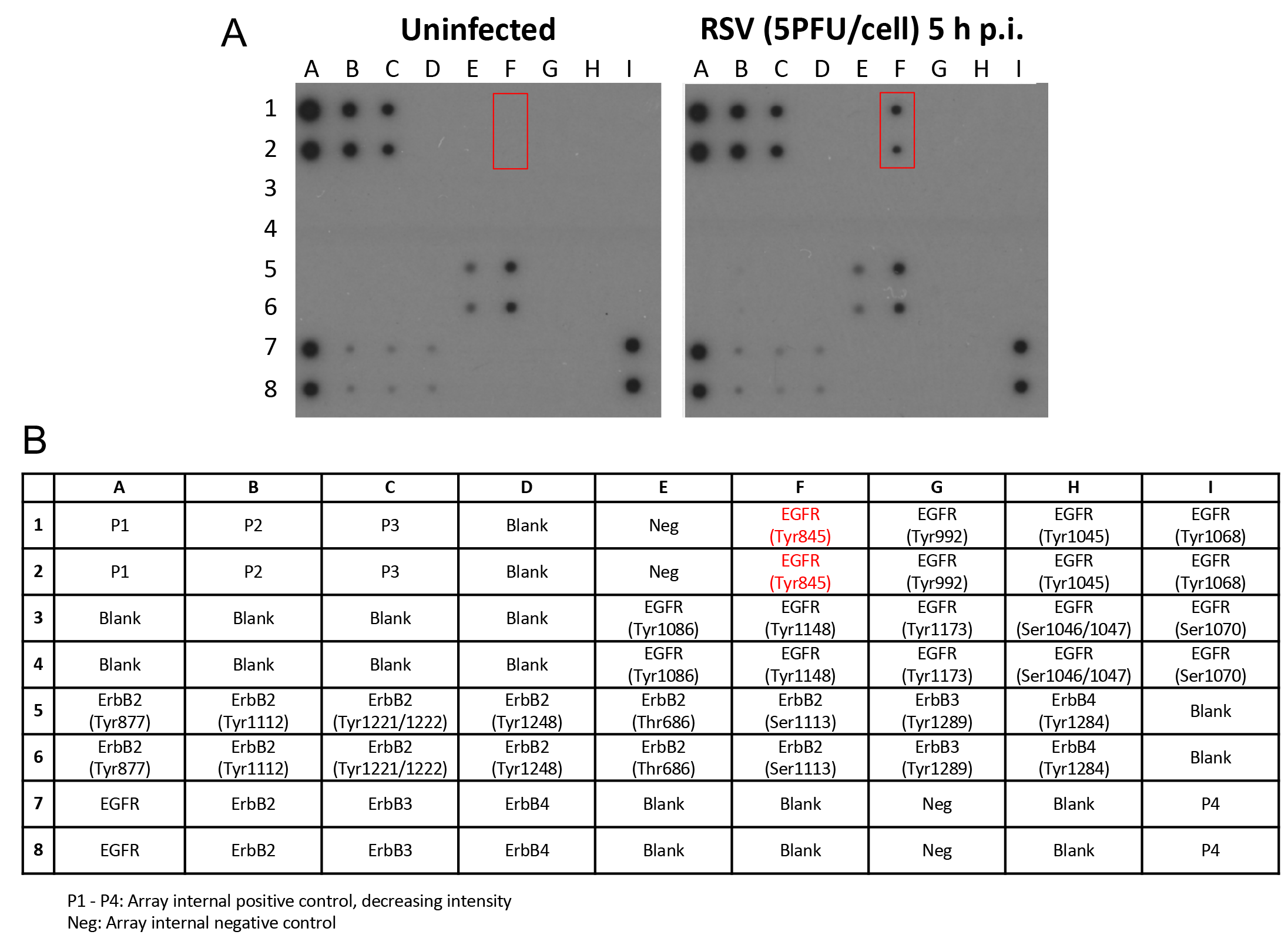

Supplement: S6 Fig — (A) X-ray films of two complete EGFR phosphorylation-specific antibody arrays, probed with uninfected (left) or RSV-infected (right) A549 cell lysates as indicated. This is from the experiment described in Fig 8, which shows selected X-ray film spots from the complete set of arrays. (B) Layout of the EGFR phospho-specific antibodies and the control spots on the array (RayBiotech). Each antibody is present in duplicate on each membrane, as shown. (TIF) [file ppat.1007963.s007.tif]

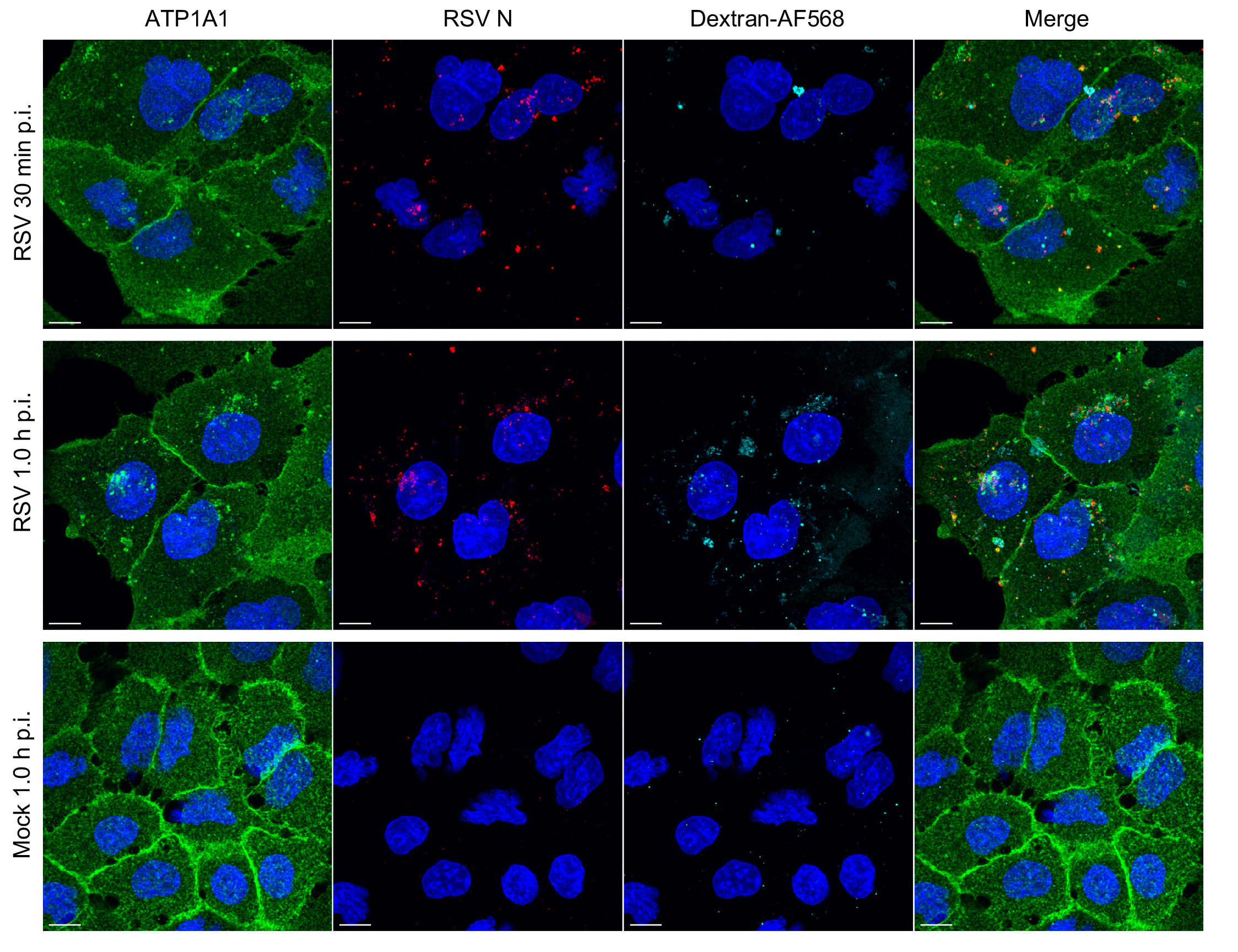

Supplement: S7 Fig — Earlier timepoints (30 min and 1 h p.i.) of the experiment shown in Fig 9B (TIF) [file ppat.1007963.s008.tif]

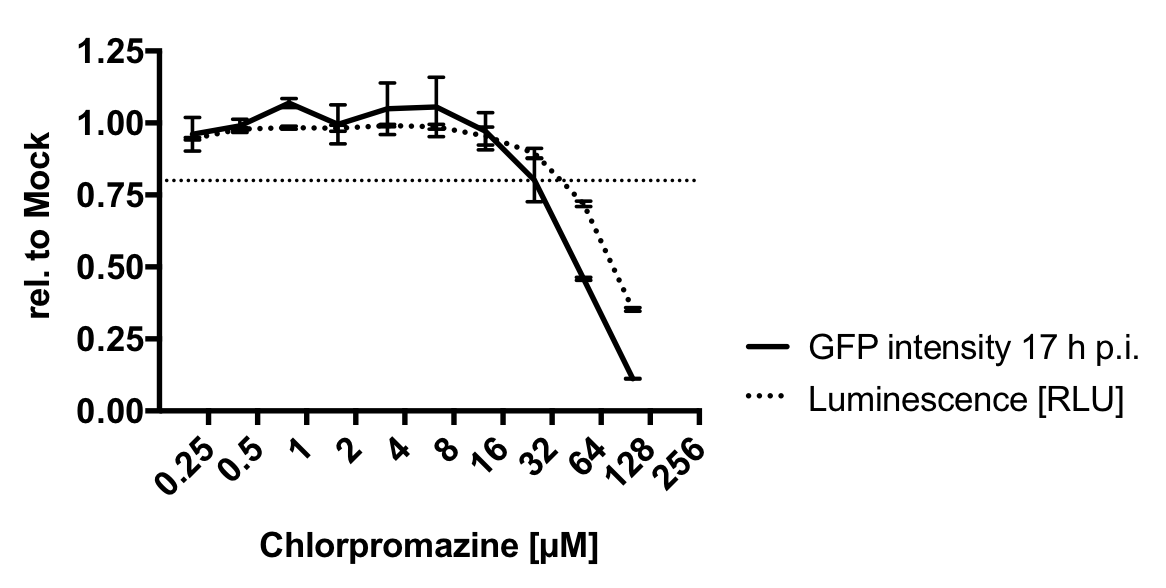

Supplement: S8 Fig — A549 cells were pre-treated for 5 h with serially-diluted concentrations of chlorpromazine and inoculated with RSV-GFP (MOI = 1 PFU/cell) while the inhibitor was continuously present. GFP was quantified by an ELISA reader 17 h p.i. (solid line) and cell viability was evaluated for each concentration by the ATP-based viability assay CellTiter-Glo (dotted line). GFP-intensity and luciferase activity was reported relative to mock-treated cells assigned the value of 1.0, with error bars indicating the SD. The dashed horizontal line reflects 80% viability. (TIF) [file ppat.1007963.s009.tif]
